# Potential Role of Flavonoids in Treating Chronic Inflammatory Diseases with a Special Focus on the Anti-Inflammatory Activity of Apigenin

**DOI:** 10.3390/antiox8020035

**Published:** 2019-02-05

**Authors:** Rashida Ginwala, Raina Bhavsar, De Gaulle I. Chigbu, Pooja Jain, Zafar K. Khan

**Affiliations:** Department of Microbiology and Immunology, and Center for Molecular Virology and Neuroimmunology, Center for Cancer Biology, Institute for Molecular Medicine and Infectious Disease, Drexel University College of Medicine, Philadelphia, PA 19129, USA; rag78@drexel.edu (R.G.); rmb345@drexel.edu (R.B.); dic26@drexel.edu (D.G.I.C.); pj27@drexel.edu (P.J.)

**Keywords:** natural products, flavonoids, apigenin, dendritic cells, neuroinflammation, chronic inflammation

## Abstract

Inflammation has been reported to be intimately linked to the development or worsening of several non-infectious diseases. A number of chronic conditions such as cancer, diabetes, cardiovascular disorders, autoimmune diseases, and neurodegenerative disorders emerge as a result of tissue injury and genomic changes induced by constant low-grade inflammation in and around the affected tissue or organ. The existing therapies for most of these chronic conditions sometimes leave more debilitating effects than the disease itself, warranting the advent of safer, less toxic, and more cost-effective therapeutic alternatives for the patients. For centuries, flavonoids and their preparations have been used to treat various human illnesses, and their continual use has persevered throughout the ages. This review focuses on the anti-inflammatory actions of flavonoids against chronic illnesses such as cancer, diabetes, cardiovascular diseases, and neuroinflammation with a special focus on apigenin, a relatively less toxic and non-mutagenic flavonoid with remarkable pharmacodynamics. Additionally, inflammation in the central nervous system (CNS) due to diseases such as multiple sclerosis (MS) gives ready access to circulating lymphocytes, monocytes/macrophages, and dendritic cells (DCs), causing edema, further inflammation, and demyelination. As the dearth of safe anti-inflammatory therapies is dire in the case of CNS-related disorders, we reviewed the neuroprotective actions of apigenin and other flavonoids. Existing epidemiological and pre-clinical studies present considerable evidence in favor of developing apigenin as a natural alternative therapy against chronic inflammatory conditions.

## 1. Introduction

Cellular inflammation can be the driving factor in many diseases, leading to either untimely cell death, causing organ-specific damage, or cell stimulation, initiating the formation of various tumors. Chronic inflammation is seen to be integral to the development of various diseases including diabetes, heart disease, cancer, digestive disorders, autoimmune diseases, or neurodegenerative disorders [1,2]. Because inflammation is the result of the immune system’s protective response to invading pathogens or endogenous signals like damaged cells, it has long been associated with the symptomatology of infectious diseases. However, a growing body of epidemiological evidence suggests that inflammation may also be linked to non-infectious diseases because of an imbalance in physiological immune responses [1,3]. According to the World Health Organization (WHO), chronic inflammation and its related diseases pose the greatest threat to public health, and a steady rise in the prevalence of such diseases is anticipated for the next 30 years in the United States alone [4]. Thus, recognizing and understanding the involvement of inflammatory processes underlining different disorders will pave the way to development of a new class of drugs to help curb the tide of chronic inflammatory diseases.

Inflammation is characterized by the protective response of the immune system that involves the recognition of highly conserved pathogenic structures (pathogen-associated molecular patterns (PAMPs)) or endogenous non-infectious molecules (damage-associated molecular patterns (DAMPs) or alarmins or cell-death associated molecules) by pathogen-recognition receptors (PRRs) [2,3,5]. The activation of these receptors leads to the production of various pro-inflammatory cytokines such as tumor necrosis factor (TNF)-α, interleukin (IL)-1β, and chemokines through the induction of the nuclear factor-kappa B (NF-κB) pathway and NLRP3 (NOD-, LRR-, and pyrin domain-containing 3) inflammasome activation. The inflammatory pathways, such as the mitogen-activated protein kinase (MAPK), Janus kinase/signal transducers and activators of transcription (JAK-STAT), and especially the NF-κB pathway, help orchestrate the inflammatory responses through the production of inflammatory cytokines and mediators, cell proliferation and survival, T-cell differentiation, and dendritic cell (DC) maturation [6]. The role of the cytokines and chemokines is to recruit additional immune cells to the site of infection, including circulating neutrophils that enhance microbial killing through the production of interferon (IFN)-γ, proteases, and reactive oxygen species (ROS). Cytokines also induce the production of cyclooxygenase-2 (COX-2), an enzyme that catalyzes the production of prostaglandins, which are key mediators of inflammation [7]. Additionally, dendritic cells, the most potent antigen presenting cells of the immune system, also aid in activating the adaptive immune response through naïve T-cell polarization and B-cell activation. Elimination of the foreign/endogenous agent and reprogramming of the effector cells to effectively end the production of inflammatory mediators then leads to resolution of inflammation and return to homeostasis. However, failure to do so leads to prolonged periods of unresolved inflammation that becomes a contributing factor in almost all chronic or degenerative diseases. Therefore, there is a need to develop therapies targeting underlying inflammation to achieve therapeutic advances targeting most of these degenerative disorders that currently have no cure.

Chronic inflammation is the leading cause of death worldwide, where three of every five individuals die as a result of chronic inflammatory diseases like diabetes, heart disorders, cancer, stroke, and obesity [4]. It constitutes a significant economic burden due to life-long debilitation leading to high therapy cost and lost wages [8,9]. Furthermore, existing therapies are rarely curative, mostly disease-modifying with low success rates, and have adverse and sometimes life-threatening side effects [10,11]. Commonly prescribed anti-inflammatory drugs include Metformin, non-steroidal anti-inflammatory drugs (NSAIDs), statins, and corticosteroids, which alleviate inflammation through several mechanisms [4,10]. Novel therapies targeting specific cells of the immune system, for example, T-cell targeted therapies (Laquinimod, Tacrolimus, Edratide), various anti-B-cell targets (Rituximab, BLyS), and cytokine inhibitors (adalimumab, infliximab), have also been employed in disease management and treatment with varying results [11,12,13,14]. However, there is still a pressing need to develop safer and cost-effective therapeutic alternatives. Natural products, which are classically defined as compounds derived from natural sources such as plants, animals, and micro-organisms, have been used for many millennia to treat a number of human ailments [15,16]. These compounds have historically served as important leads for pharmaceutical companies in the development of synthetic drugs, which were initially produced in the form of crude compilations and more recently, with the advent of combinatorial chemistry and sophisticated techniques like genomics and proteomics, consist of purified compounds [17,18,19,20]. Also, synthetic derivates of natural compounds with certain enhanced characteristics can also be engineered. In fact, about 34% of the U.S. Food and Drug Administration (FDA)-approved medicines between 1981 and 2010 are natural products or derivatives of natural products including anti-cancer drugs and immunosuppressants [21]. Although, combinatorial chemistry has also resulted in the relative ease with which synthetic libraries of small molecule drugs can be generated, scaling back natural product-based drug discovery [22,23]. However, seminal discoveries like that the first naturally derived medicine, morphine, to those of penicillin and streptomycin and the more recent anti-parasitic drugs avermectins and artemisinin, show that natural products are definitely the best source of drugs [24,25]. With newer techniques comes the knowledge of the structures of the different natural compounds, which in turn allows for understanding their specific mechanism of action in health and disease.

As seen with their role in the treatment of several diseases including malaria, cancer, and diabetes, natural products have been reported to have inhibitory effects on inflammation. In the past several decades, numerous studies have reported the anti-inflammatory activities of various plants, plant extracts, or purified compounds derived from plant and other natural sources [26,27,28]. Various plant-derived compounds including curcumin, resveratrol, and capsaicin inhibit inflammation through reduction in the levels of several cytokines including IL-1β, IL-6, and TNF-α, and the suppression of COX-2, prostaglandins, and inflammatory pathways [29]. Active organosulphur compounds in garlic such as ajoene, alliin, and allicin work by reducing levels of pro-inflammatory cytokines while increasing levels of anti-inflammatory IL-10 [30]. Natural products derived from marine flora including those of coral and algal origin also inhibit inflammation through suppression of IL-6, TNF-α, and nitric oxide (NO) release and inhibition of COX-2, inducible NO synthase (iNOS), and NF-κB activity [31,32]. Another group of plant-derived natural products is the polyphenolic bioactive components of various plants and vegetables known as ‘flavonoids’. The word flavonoid is a derivative of the latin word *flavus*, meaning ‘yellow’, indicating the color of these compounds in their natural form [33,34,35]. Flavonoids, as secondary metabolites in numerous fruits, herbs, root, stems, bark, flowers, grains, tea, and wine, impart both color and protection to the plants and make them safe for consumption, for which they are also termed as ‘phytonutrients’. Because of their broad spectrum of biological activity and attractive properties such as anti-oxidant, anti-mutagenic, anti-inflammatory, and anti-viral effects, these compounds present an indispensable library of compounds that can be developed as therapeutic entities. This review will focus on the anti-inflammatory activities of various flavonoids that can potentially work against several chronic diseases. We will then briefly showcase the known anti-inflammatory properties of a comparatively less-toxic flavonoid, apigenin, to assess its potential as a drug lead against chronic neuroinflammatory diseases. 

## 2. Flavonoids in Health and Disease

Flavonoids are a multi-functional group that possess substantial characteristics that can be exploited for the development of therapeutic agents targeting several chronic diseases. They have been seen to exert a wide range of pharmacological effects, such as anti-oxidant, anti-tumor, anti-viral, anti-allergic, anti-inflammatory, and anti-viral effects. These protective biological properties are mostly due to the phenolic structure of these flavonoids.

### 2.1. Chemical Structure

Flavonoids belong to the group of polyphenolic natural compounds, with more than 4000 identified varieties. This variation is the result of the modifications that are possible to the carbon skeleton, which is common to all flavonoids and consists of a flavan system of two benzene rings (denoted as A and B) that are linked together by a heterocyclic pyrene ring (denoted as C) [36]. The chemical diversity of flavonoids is based on two structural variations; namely the pattern of substitution of the C ring that depends on the carbon on which the B ring is attached, and the degree of oxidation of the C ring [33,37]. The basic flavonoid structure is aglycone, but they typically occur in nature as glycoside and methylated derivatives, which are products of secondary metabolism in plants [36].

Certain structural modifications can also favor the anti-inflammatory activities of the flavonoid families. Current understanding of the structural requirements dictates that the unsaturation of the C ring, the presence of a carbonyl group on C-4, the number and position of hydroxyl groups, and glycosylation status affect the anti-inflammatory properties of flavonoids [38,39]. For example, the presence of a catechol group in the B ring of the flavonoid quercetin confers potent anti-inflammatory activity, while the addition of one hydroxyl group on position 2′ of the B ring of morin abolishes any anti-inflammatory properties [38]. Additionally, the hydroxylation pattern of the B ring of certain flavonoids promotes the inhibition of cytokine secretion by mast cells and macrophages [40]. Glycosylation of flavonoids has been linked to a reduction in the inhibitory effect on inflammation because glycoside derivatives are more readily absorbed than aglycones. 

### 2.2. Subclasses

On the basis of the molecular structure, flavonoids can be divided into different subclasses (as described in detail in Table 1) as follows: flavonols (e.g., quercetin, kaempferol, myricetin, and fisetin), flavones (e.g., apigenin and luteolin), flavanones (e.g., hesperetin and naringenin), flavononols, anthocyanidins, and isoflavones [33,36]. The various compounds within a specific subclass differ in the pattern of substitution of the A and B rings. The process of flavonoid biosynthesis is conserved in plant, where the action of enzymes modifies the basic flavonoid structure, leading to various intermediary compounds or flavonoid subclasses. The C ring of the flavonoid precursors, chalcones, closes to form a chromone unit, resulting in the formation of flavanones. Oxidation at the third carbon of flavanones produces the flavanols. Flavones are then formed by a double bond at C-2. The reduction of flavonols produces flavan-3-ols such as anthocyanidins [38]. These subclasses have varying distribution among the different natural sources, for example, flavonols and flavones are typically abundant in onions and tea.

### 2.3. Health Benefits

A wide range of biological activities have been attributed to the flavonoid group of natural products. These include anti-oxidant, anti-inflammatory, anti-mutagenic, anti-viral, and anti-allergic properties. Owing to their structure, flavonoids exert various protective effects against several chronic diseases like cancer, diabetes, and cardiovascular disorders, as well as neurodegenerative conditions. By complexing with oxidizing species, hydroxyl groups in flavonoids render these compounds the ability to scavenge and stabilize free radicals, reducing oxidative damage, which is the hallmark of several chronic diseases [36,41]. Here, we review the effects of flavonoids on various inflammatory processes that cause or further exacerbate chronic diseases.

### 2.4. Flavonoids in Diseases of Chronic Inflammation

Flavonoids comprise a wide variety of biologically active compounds, many of which have been used as components of various medicinal preparations over thousands of years to treat several human illnesses. Most of the non-infectious diseases develop or are considerably worsened by the presence of persistent chronic inflammation. Here, we will explore the characteristic functions of flavonoids that help combat many inflammatory processes underlying several chronic conditions such as cancer, obesity, and neuroinflammation.

#### 2.4.1. Flavonoids in Cancer

German pathologist, Rudolf Virchow, was the first to find a link between inflammation and cancer development. Since then, epidemiological studies have established a correlation of at least 20% of cancers, including lung, prostrate, bladder, pancreatic, esophageal, and melanoma, with long-term inflammation [42,43]. Chronic unregulated inflammation results in the persistent production of harmful ROS that can lead to DNA damage and genomic alterations, causing the initiation of tumor growth. There is also a continuous generation of inflammatory mediators such as IFN-γ, TNF, IL-1α/β, or IL-6 and proangiogenic growth factors such as cytokines and vascular endothelial growth factor (VEGF) that promote tumor neovascularization, which brings the much-needed blood supply, nourishing the growing tumor. Finally, inflammation promotes tumor dissemination through the production of extracellular matrix degrading enzymes, the matrix metalloproteinases (MMPs) [44]. All these factors are either produced by tumor infiltrating immune cells such as macrophages, dendritic cells, neutrophils, lymphocytes, and natural killer cells or by the cancer cells themselves to stimulate their growth and survival. The key inflammatory pathway, NF-κB, plays an important role in cancer cell survival by allowing these cells to escape apoptosis [45]. Consequently, therapeutic agents that target these inflammatory factors and pathways will serve as a means to treat or prevent cancer development. 

**Figure 1:**
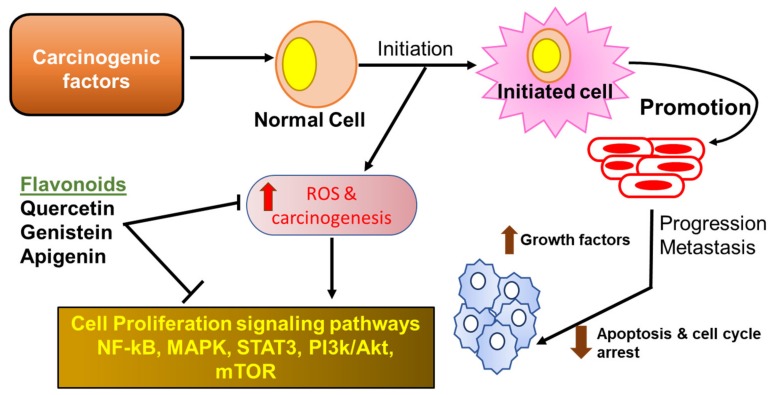
Flavonoids in cancer. Flavonoids exert their anti-inflammatory activities by reducing the production of reactive oxygen species (ROS) and the down-regulation of several inflammatory mediators through key inhibition of signaling pathways. NF-κB—nuclear factor-kappa B; MAPK—mitogen-activated protein kinase; STAT—signal transducers and activators of transcription.

Because flavonoids possess several anti-inflammatory properties, they can serve as potent anti-cancer phytochemicals (Figure 1) that exert their activity by several mechanisms such as carcinogen inactivation, triggering cell cycle arrest, induction of apoptosis, and inhibition of angiogenesis [46,47,48,49,50]. Flavonoids have been shown to inhibit tumor cell proliferation via inhibition of ROS formation, as well as suppression of xanthine oxidase, COX-2, and 5-LOX, which are the major catalysts for tumor promotion and progression (reviewed in the work of [51]). Mojzis et al. reported that flavonoids are able to exert anti-angiogenic effects by regulating the expression of VEGF, MMPs, and epidermal growth factor receptor (EGFR), as well as by inhibiting the proangiogenic signaling pathways including the NF-κB, PI3-K/AKt, and ERK1/2 pathways [52]. Accumulating evidence reported that cyclin dependent kinases (CDKs) are key regulators of cell cycle progression, immune cell activation, neoangiogenesis, and inflammation [53]. In addition, it has been seen that various types of cancers are associated with hyper-activation of CDKs, because of mutation of CDK genes or CDK inhibitor genes. Flavonoids have been shown to induce cell cycle arrest at both checkpoints G1/S and G2/M through CDK inhibition in human breast cancer and melanoma cells [51]. Flavonoids such as isoflavones and their metabolites also induce cancer cell apoptosis in cells derived from human gastric cancer [50] by inhibiting DNA topoisomerase I/II activity, decreased production of ROS, regulation of heat shock protein expression, modulation of signaling pathways, suppression of NF-κB, activation of endonuclease, and suppression of Mcl-1 protein. 

#### 2.4.2. Flavonoids in Diabetes

Inflammation has long been associated with the promotion of type 1 diabetes, however, more recently, chronic low-grade inflammation has been deemed responsible for onset and/or exacerbation of type 2 diabetes, diabetes mellitus [54]. Type 2 diabetes is associated with impaired insulin secretion and insulin resistance. Nutrient excesses such as hyperglycemia and elevated free fatty acids, as seen in type 2 diabetes, cause oxidative stress, endoplasmic reticulum stress, amyloid and lipid deposition, lipotoxicity, and glucotoxicity induced by inflammatory processes. Metabolic dysregulation associated with diabetes is said to induce a proinflammatory response in macrophages residing in the adipose tissue, islets, and vasculature, and their infiltration into the adipocytes is directly proportional to cell size [55]. Cellular stresses are induced by the activation of thioredoxin-interacting protein and NLRP3 inflammasome, releasing increased amounts of IL-1β, contributing to β-cell dysfunction and insulin resistance [56]. Macrophages, endothelial cells, and adipocytes also produce acute-phase proteins such as C-reactive protein (CRP) and serum amyloid A (SAA) in response to elevated levels of IL-6. Additionally, proinflammatory cytokine TNF-α released within the adipose tissue promotes lipolysis and increases free fatty acids [57].

Several studies have reported the beneficial properties of flavonoids that promote their use as a supplementary treatment for diabetes mellitus [58]. It has been seen that flavonoids can modulate carbohydrate and lipid metabolism, attenuate hyperglycemia, insulin resistance, alleviate oxidative stress and stress-sensitive signaling pathways, and inflammatory processes [59]. Flavonoids morin, hesperidin, rutin, (rats), and chrysin (mice) were effective in reducing inflammatory cytokines IL-1β, IL-6, and TNF-α in diabetic animals, significantly improving hyperglycemia, glucose intolerance, and insulin resistance [60,61,62,63]. Diabetes mellitus can eventually cause secondary damage to various organs of the affected individual such as eyes, kidneys, nerves, and heart [64]. Flavonoids not only help restore glucose homoeostasis attenuating the diabetic condition, but also regulate the secondary damage to the various peripheral organs. Hesperidin reversed neuropathic pain and improved heart function in diabetic rats [63,65]. Chrysin improves cognition, prevents the development of diabetic neuropathy, and improves renal pathology in diabetic rats [66,67].

Peroxisome proliferator-activated receptor gamma (PPAR-γ), a nuclear receptor found in adipocytes and macrophages, stimulates adipogenesis, lipid uptake, and inulin sensitivity and regulates inflammation and glucose metabolism through upregulation of adipokines and glucose transporter, GLUT4, as well as fatty acid binding and transport proteins, respectively [68,69]. Certain flavonoids such as naringenin, luteolin, and quercetin alter PPAR-γ activation and increase insulin sensitivity [70]. PPAR-γ also induces various adipokines including leptin, adiponectin, and resistin, which play crucial roles in glucose homeostasis and regulate inflammation [70]. Adiponectin has been seen to inhibit the release of pro-inflammatory cytokines such as TNF-α and IL-6. Luteolin, abundant in vegetables and fruits including celery, parsley, carrots, and apple skins, potentiates insulin action and increases expression and transcriptional activation of PPAR-γ and expression of the PPAR-γ target genes, increasing expression of adiponectin, leptin, and GLUT4 in 3T3-L1 adipocytes, as well as in primary mouse adipose cells [71]. Liu et. al. reported that the decrease in circulating level of inflammatory molecules MCP-1, resistin, and the elevation of adiponectin level in obese mice may be attributed to luteolin, which, in turn, mediates beneficial effects on metabolic pathways implicated in insulin resistance and type 2 diabetes pathophysiology [72].

#### 2.4.3. Flavonoids in Inflammatory Bowel Disease

Inflammatory bowel disease (IBD) is characterized by chronic and uncontrolled gastrointestinal inflammation, associated with both impaired epithelial mucosal barrier and altered innate and adaptive immune responses [73,74]. IBD presents as two main clinical and pathological subtypes, Crohn’s disease (CD) and ulcerative colitis (UC). Both forms of IBD result in poor quality of life and require prolonged medical and/or surgical interventions. CD can affect any part of the gastrointestinal tract, from the mouth to the anus, but it is usually, although not always, localized in the distal small bowel and/or colon. In contrast, UC is restricted to the colon and the rectum. Although several theories have been put forth to explain IBD pathogenesis, the exact cause of IBD still remains elusive [73,75]. IBD patients who exhibit a dysfunctional intestinal epithelium barrier with higher tight junction permeability develop an exaggerated immune response in the gut towards the intestinal microbiota. Several elements of the mucosal immune system including intestinal epithelial cells, innate immune cells such as dendritic cells, macrophages, neutrophils, and cells of the adaptive immune system such as T cells and B cells, along with cytokines and chemokines, have been shown to participate in the pathogenesis of IBD [75]. Tolerance to commensal flora in IBD is lost as a result of dendritic cell overactivation and the resultant loss of regulatory T cells and strong induction of proinflammatory effectors such as T_h_1, T_h_17, and natural killer (NK) cells [76,77]. Additionally, a variety of cell adhesion molecules (CAMs), including intracellular CAM-1 (ICAM-1) and vascular CAM (VCAM-1) and chemokines such as IL-1β, IL-6, and TNF-α, have been shown to be activated by endothelial cells, which are responsible for the recruitment of leukocyte and promoting inflammatory responses [78].

As the exact etiology of IBD is not well understood, there is no specific treatment available to cure the disease. Hence, flavonoids, which are known to have a range of biological activities, could be beneficial in the treatment for IBD [79,80,81]. Flavonoids such as apigenin and epigallocathechin gallate have been shown to inhibit the activation of immune cells and the downstream chemokines and cytokines [82], thereby it may considered as a natural inhibitor and can prevent the activation of an innate and adaptive immune system [79]. A number of studies have published the anti-inflammatory impact of flavonoids in several experimental models of colitis (reviewed in the work of [75]). Flavonoids belonging to different subclasses such as chalcones (cardamonin), isoflavones (genistein, daidzein, glabridin), anthocyanidins (cyanidin-3-glucoside (C3G)), flavonols (quercetin, quercitrin, rutin), flavanones (naringenin), flavones (baicalin, chrysin), and catechins (epigallocatechin-3-gallate) have shown profound intestinal anti-inflammatory activity. Of these, accumulating evidence has shown that quercetin inhibits bacterial lipopolysaccharide (LPS)-induced iNOS and TNF-α secretion in macrophages, LPS-induced IL-1β, TNF-α secretion in RAW2647 and cytokine induced expression of VCAM-1 and ICAM-1, and E-selection in HUVECs (reviewed in the work of [83]). Glycones have been more effective in curbing intestinal inflammation as they are metabolized in the colon, where the aglycone form is then released as opposed to the early absorption of aglycones in the intestines. Quercetin glycosides quercetrin and rutin have more potent effects on disease outcomes than the aglycone [84,85]. Quercetrin reduces colonic inflammation in rats through modulation of the NF-kB pathway and subsequent inhibition of cytokines, as well as iNOS in vivo [86]. Naringenin (4’,5,7-trihydroxy avanone-7-rhamnoglucoside), which belongs to the flavanone class, is shown to attenuate the severity of colitis by inhibiting myeloid derived suppressor cells (MDDCs), pro-inflammatory mediators, and the NF-kB/IL-6/STAT-3 cascade in colorectal tissues [87].

#### 2.4.4. Flavonoids in Non-Alcoholic Fatty Liver Disease

Non-alcoholic fatty liver disease (NAFLD) is rapidly emerging as the most common etiological factor for chronic liver disease, largely because of the growing prevalence of insulin resistance, obesity, and diabetes [88,89]. As the name suggests, NAFLD is characterized by the deposition of free fatty acids and triglycerides in hepatocytes and can lead to further complications such as liver fibrosis, cirrhosis, and hepatocellular carcinoma [89,90]. NAFLD comprises a spectrum of disorders including simple steatosis in the absence of inflammation and hepatocellular damage and the more severe form of NAFLD, nonalcoholic steatohepatitis (NASH), characterized by lobular inflammation [91]. Much remains to be elucidated about the pathogenesis of both NAFLD and NASH, however, attempts to depict a ‘two-hit’ hypothesis of disease pathogenesis have been made. Hepatic steatosis due to deposition of triglycerides coming from lipolysis of adipose tissue, from de novo synthesis, or from the diet triggered by insulin resistance constitutes the first ‘hit’ [90,92]. Excessive accumulation of fat and the presence of circulating free fatty acids contribute to oxidative damage, immune system activation, and dysregulation of cytokine pathways through recognition by PRRs such as toll-like receptors (TLR)s, in particular, TLR4 [92]. Lipid accumulation in the liver leads to increased transcription and the release of IL-6, TNF-α, and C-reactive protein, leading to chronic low-grade inflammation through the reduction of the anti-inflammatory adiponectin, which sensitizes hepatocytes to insulin [90].

Because the pathogenesis of NAFLD is multifaceted, so far there is no evidence-based treatment for this disease. In this aspect, bioactive compounds such as flavonoids that can modulate various pathways are ideal candidates for therapeutic development against NAFLD. Flavonoids have been shown to have beneficial effects on lipid metabolism, insulin resistance, oxidative stress, and inflammation, the major causative factors of NAFLD [93]. The anti-inflammatory effects of silymarin, a flavonoid mix derived from milk thistle, have been documented in animal models of NAFLD [94]. Silibinin, the most active component of silymarin, decreased hepatic NF-κB activation and decreased levels of ROS and iNOS in a mouse model of NASH [95]. Silymarin was also reported to have reduced the expression of inflammatory TNF-α mRNA in the liver of methionine- and choline-deficient (MCD) diet induced NASH in insulin-resistant rats [96]. Isoflavones found in soybeans and their derivatives have also shown beneficial effects on NAFLD in in vivo animal studies. Genistein, a soy phytoestrogen, has been reported to show anti-inflammatory effects through reduction of TNF-α and IL-1β mRNA expression in nonalcoholic fatty liver disease db/db mice [97]. Genistein administration in NASH rats induced by a high fat diet alleviated liver damage through inhibition of inflammatory processes by reducing serum levels of TNF-α and IL-6 and inhibiting IκB-α phosphorylation, nuclear translocation of NF-κB p65 subunit, and activation of c-Jun N-terminal kinase (JNK) [98]. C57BL/6 mice on a cholesterol-enriched diet supplemented with the isoflavone, 2-heptyl-formononetin, demonstrated lowered hepatic inflammation through a reduction in TNF-α levels and macrophage infiltration [99]. Both quercetin and its glycoside rutin showed reduction in inflammatory markers TNF-α and IL-6 in NASH mice [100,101]. Other flavonoids such as cyanidin 3-O-β-D-glucoside and xanthohumol also inhibit inflammatory pathways through ROS inhibition and suppression of NF-κB and its dependent genes in animal models [102,103].

#### 2.4.5. Flavonoids in Cardiovascular Disorders

Inflammatory mediators are both a predictive and a causative factor in the pathogenesis of cardiovascular disorders. Acute myocardial infarction (AMI) is the result of rupture of an atherosclerotic plaque, leading to thrombus formation and loss of blood flow causing ischemia in the area distal to the occlusion [104]. Prolonged myocardial ischemia leads to cardiomyocyte injury releasing DAMPS, which activate platelets and leucocytes; recruit neutrophils; and lead to endothelial cell injury and the production of ROS, proteases, and cytokines. Additionally, the NLRP3 inflammasome is activated in myocardial ischemia, which in turn binds to and activates caspase 1, which is responsible for the conversion of IL-1β to its active form. Of all the cytokines, TNF-α, IL-1β, and IL-6 play central roles in AMI, causing the secretion of other cytokines, chemokines, and adhesion molecules augmenting further leucocyte infiltration. Similarly, immune cells and released mediators also play a critical role in the initiation and progression of atherosclerosis [105]. Plaque formation is initiated by the accumulation of low-density lipoproteins (LDLs) in the subendothelial layers of the arteries, leading to endothelial injury and dysfunction. This subsequently leads to formation of ROS, which oxidize the LDLs and contribute to plaque formation. Activated endothelial cells further release leucocyte adhesion molecules such as vascular cell adhesion molecule-1 (VCAM-1), intercellular adhesion molecule 1 (ICAM-1), and selectins, which, together with chemokines such as CCR2, and CCR5, recruit circulating monocytes. Monocytes then differentiate in situ to macrophages that convert to foam cells via uptake of oxidized LDLs and release a milieu of cytokines such as TNF-α, IL-1, IL-3, IL-8, and IL-18.

Manipulation of these inflammatory events is crucial in the prevention and management of cardiovascular diseases. Because of the phenol hydroxy groups present in flavonoids, these compounds possess remarkable anti-oxidant and free-radical scavenging properties. Thus, these polyphenols are able to show promising effects in the management of cardiovascular injury. In vitro evidence shows that quercetin reduces LDL oxidation at physiological levels in human umbilical vein endothelial cells [106]. Similar reduction in macrophage-mediated LDL oxidation was seen after treatment with fisetin and proanthocyanidins [107]. Quercetin was also shown to decrease the level of proinflammatory mediators such as TNF-α, IL-6, MIP-1α, and P-selectin in murine RAW264.7 macrophages [108]. Further, quercetin treatment can potentially disrupt atherosclerotic plaques through the inhibition of matrix metalloproteinase 1 [109]. Soy isoflavone administration reduced the risk of chronic inflammation-mediated cardiovascular disease by reducing the endothelial production of TNF-α in a mouse model [110]. Isoflavones have also been reported to protect against inflammatory vascular disease through the inhibition of monocyte recruitment across the endothelium. Additionally, several flavonoids such as apigenin, chrysin, and kaempferol have been shown to inhibit the expression of adhesion molecules on human aortic endothelial cells, thereby limiting leucocyte infiltration [111].

#### 2.4.6. Flavonoids in Neuroinflammation

Neuroinflammation is the accompanying factor in several neurodegenerative diseases such as Alzheimer’s disease (AD), Parkinson’s disease (PD), Huntington’s disease (HD), and multiple sclerosis (MS). These neurodegenerative diseases result in the progressive and irreversible loss of neurons in the brain [112]. Activation of central nervous system (CNS)-resident microglial cells is one of the crucial events in the inflammatory cascade, which leads to the progression of neurodegeneration [113]. Prolonged activation of microglial cells may contribute to neurodegeneration through the release of pro-inflammatory mediators such as prostaglandins, NO, TNF-α, IL-6, and IL-1β, resulting in chronic CNS neuroinflammation [114]. These inflammatory events contribute to the apoptotic cell death of neurons in many neurodegenerative diseases. Additionally, encephalitogenic inflammatory CD4+ T cells such as T_h_1, T_h_17, granulocyte macrophage colony-stimulating factor (GM-CSF)-producing CD4+ T-cells, and γδT-cells play very crucial roles in the initiation and propagation of neuroinflammation in autoimmune diseases such as MS. T cells become activated by antigen presenting cells (APCs) such as dendritic cells (DC) and macrophages. DCs are the most efficient professional APCs and play an important role in various autoimmune and neuroinflammatory diseases [115,116,117]. Our earlier studies have reported that DCs can migrate into diverse regions of the CNS [118] in response to neuroinflammatory signals (i.e., chemokine CCL2) both in vitro and in vivo [119]. Additionally, increased frequencies of both plasmacytoid and myeloid DCs (pDC and mDC) have been reported in the cerebrospinal fluid (CSF) of MS patients [120]. In fact, CD11c^+^ DCs were shown to be sufficient to present myelin antigen to naive T cells, leading to the development of experimental autoimmune encephalomyelitis (EAE) in mice [121]. Hence, therapeutic agents targeting DC function and their migration across the inflamed blood–brain barrier (BBB) under neuroinflammatory conditions will be of vital importance in the treatment of neurodegenerative diseases such as MS. The treatments currently available are rarely curative and have serious side effects.

Natural flavonoids have been shown to exert neuroprotective properties by inhibiting the release of pro-inflammatory cytokines. Flavonoids exert an anti-inflammatory effect via interfering with the development of inflammatory mediators such as IL-6, TNF-α, and IL-1β in several cell lines through the MAPK signaling pathway [122]. TNF-α and iNOS expression has been seen to be regulated by inhibiting MAPK signaling cascade molecules such as p38 or ERK1/2. Accumulating evidence has reported that flavonoids can modulate the activity of various metabolic pathways to reduce neuronal dysfunction. Moreover, flavonoids have been found to delay or prevent the onset of neurodegenerative diseases at their effective doses in various animal models [123]. Wogonin, baicalein, curcumin, apigenin, quercetin, luteolin, and many other flavonoids have been shown to exhibit neuroprotective effects. Wogonin and baicalein have been shown to exert various anti-inflammatory effects through the inhibition of inflammatory microglia. It has been seen that wogonin can inhibit the LPS induced production of NO, inducible NO synthase (iNOS), and NF-κB activation in microglia. LPS induced NF-κB activity in BV-2 microglial cells was shown to be inhibited by the flavonoid baicalein without interfering activation of caspase-11, activator of transcription (STAT-1), and induction of interferon regulatory factor (IRF-1) [124]. Further, curcumin inhibits the apoptosis of pre-oligodendrocyte mediated expression of iNOS, NO, and COX-2 in LPS activated microglia [125]. Luteolin exerts its immunomodulatory effects on peripheral blood mononuclear cells (PBMC) derived from MS patients. Luteolin has been seen to suppress the PBMC production of several pro-inflammatory cytokines such as IL-1β, metalloproteinase-9 (MMP-9), and TNF-α, which play very crucial roles in the pathogenesis of MS [126]. Isoflavones such as daidzein and genistein inhibit TNF-α, IL-1, IL-6, iNOS, and COX-2 via suppression of ERβ and NF-κB, respectively, in primary astrocytes. Several flavonoids reportedly suppress the inflammatory activity of DCs. Luteolin inhibits LPS-induced NF-κB signaling through the suppression of IκB kinase activity in murine bone-marrow derived DCs. Flavonoids such as silibinin, taxifolin, and epigallocatechin inhibited the immunostimulatory effects of DCs via different mechanisms such as suppression of MAPK, impaired p65 translocation, and endocytic ability [127,128,129,130]. However, the effects of flavonoids on DC function in the context of neuroinflammatory diseases are relatively unknown. 

Apigenin, a polyphenolic flavonoid, abundant in chamomile plant and also found in other sources such as parsley, celery, and grape fruit, is a relatively less toxic and non-mutagenic compound among the various flavones. Apigenin can also cross the blood–brain barrier (BBB) and has been shown to exert anti-inflammatory effects on BV-2 and primary microglial cells through inhibition of p38 and JNK. Apigenin prevents neuronal apoptosis by protecting the neurons against inflammatory stresses [131]. The anti-inflammatory and neuroprotective effects of apigenin have not yet been extensively characterized. Utilizing EAE models of MS, we recently observed a significant reduction in disease severity accompanied by an increased retention of immune cells in the periphery upon treatment with apigenin [132]. These results were supported by decreased immune cell infiltration and reduced demyelination in the CNS of the apigenin-treated EAE mice. We hypothesized that the neuroprotective effects of apigenin in EAE were due to its inhibition of DC phenotypical and functional maturation and its subsequent polarization of CD4 T helper cells (unpublished data). Because of its relatively long half-life, delayed plasma clearance, and slow metabolism in the liver [133], apigenin has considerable potential to be developed as a safer, more cost-effective treatment for neurodegenerative diseases. Here, we shall review the anti-inflammatory properties of apigenin that have been extensively characterized in various disease systems and debate its potential as a therapeutic drug candidate for neuroinflammatory conditions like MS.

## 3. Role of Apigenin as an Anti-Inflammatory Agent

**Figure 2:**
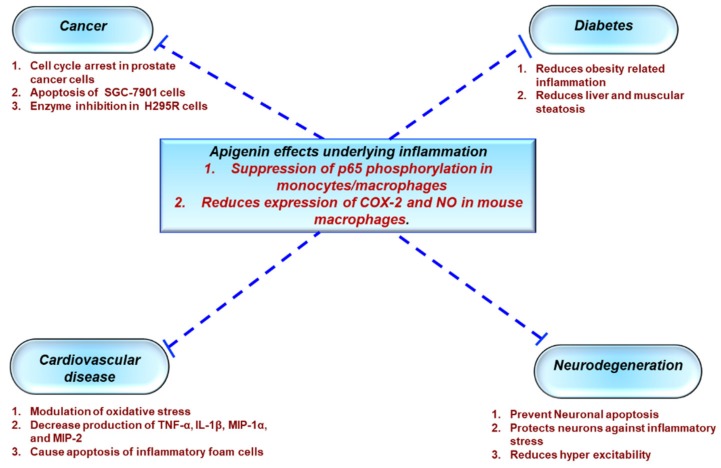
Role of apigenin in chronic inflammatory diseases. Apigenin as an anti-inflammatory compound acts as a protective agent in several disorders via inhibition of key inflammatory mediators, signaling pathways, and molecules. COX-2—cyclooxygenase-2; IL—interleukin; TNF—tumor necrosis factor; NO—nitric oxide.

As the health effects of polyphenols depend on intake and bioavailability, the biological activities of apigenin (4’,5,7-trihydroxyflavone), an abundantly occurring flavone have, been extensively studied [134,135]. Much like the family to which it belongs, apigenin possesses a wide array of biological properties including anti-oxidant, anti-cancer, and anti-inflammatory actions [136,137,138]. As a result, apigenin has gained a lot of interest in the past few years as a potential therapeutic agent to treat various diseases such as cancer, diabetes, cardiovascular, and neurological disorders [139] (Figure 2).

### 3.1. Protective Effects of Apigenin Across a Spectrum Of Chronic Diseases

According to the National Cholesterol Education Program’s Adult Treatment Panel III (NCEP: ATP III), metabolic syndrome is associated with abdominal obesity, dyslipidemia, hyperglycemia, inflammation, insulin resistance, or diabetes mellitus, as well as an increased risk of developing cardiovascular disease. Obesity is attributed to adipose tissue dysfunction and expanded adipose tissue mass, which can lead to the upregulation of proinflammatory cytokines such as TNF-α and IL-6, resulting in a state of chronic low-grade inflammation, as previously discussed. Apigenin has been shown to inhibit an important inflammatory biomarker, CD38, in a metabolic syndrome model, as well as decrease adipose tissue mass and the levels of proinflammatory cytokines [140]. Additionally, Feng et al. have reportedly shown that apigenin improves obesity and obesity induced inflammation [141]. Apigenin has also been reported to attenuate inflammation and the resultant pathological alterations in rats fed with a high fat, fructose diet [142]. In diabetic rats, apigenin reduces metabolic inflammation by successfully polarizing infiltrating macrophages to an anti-inflammatory M2 phenotype by binding and activating PPAR-γ and the subsequent suppression of the NF-κB pathway [141]. Apigenin also ameliorated renal dysfunction in diabetic rats by suppressing inflammation through reduced secretion of TNF-α and IL-6 via MAPK inhibition. Histopathology confirmed reduced inflammation in the renal tissue along with reduction in collagen deposition and glomerulosclerosis [143].

Apigenin served as a potent therapy against UC in C57BL/6 mice through the inhibition of inflammatory cytokines,and COX-2, and through the reduction in immune cell infiltration in colon tissues [144]. Because NF-κB activation upregulates epithelial cell permeability, promoting colonic inflammation, testing apigenin effect in vitro on colon carcinoma cells HCT-116 demonstrated NF-κB downregulation in a dose-dependent manner. Apigenin also reduced levels of matrix metalloproteinase (MMP-3), which aids in extracellular remodeling, contributing to colonic inflammation, thereby showing protective effects in a murine DSS (dextran sulphate sodium) colitis model [145]. The use of a soluble form of apigenin showed amelioration of in colitis models in rats through the inhibition of various inflammatory markers such as TNF-α, transforming growth factor-b, IL-6, intercellular adhesion molecule 1, or chemokine (C–C motif) ligand 2 [146].

Inflammation in NAFLD is one of the main causes of insulin resistance with inflammatory markers such as TNF-α and IL-6 suppressing insulin receptor signaling, thus blocking the action of insulin in hepatocytes. In NASH mice fed with a high fat diet, apigenin ameliorated inflammation through reduction of plasma levels of MCP-1, IFN-γ, TNF-α, and IL-6 [147].

Beneficial aspects of apigenin activity help to ameliorate inflammation-mediated cardiac injury, indicating a role for apigenin as a therapeutic agent against cardiovascular diseases. In an LPS-induced model of myocardial injury, apigenin relieved injury by modulating both oxidative stress and inflammatory cytokines such as TNF-α, IL-1β, MIP-1α, and MIP-2 through NF-κB regulation [148]. Macrophages loaded with oxidized LDLs contribute significantly towards the progression of atherosclerotic plaques. Apigenin was shown to induce apoptosis of murine peritoneal macrophages through reduction in expression of anti-apoptotic plasminogen activator inhibitor 2 [149]. Apigenin can promote apoptosis in foam cells through inhibition of autophagy and subsequently reduce the foam-cell mediated secretion of proinflammatory cytokines during atherogenesis [150]. Additionally, apigenin helped in cholesterol efflux from macrophages in atherosclerotic lesions in apoE^−/− ^mice challenged with LPS through the increased expression of ATP binding cassette A1 (ABCA1) and reduced expression of proinflammatory cytokines, and reduced levels of NF-κB and TLR-4 [151].

Several studies have investigated the anti-cancer effects of apigenin and shown its ability to suppress cancer cell proliferation in various types of tumors, including pancreatic, colorectal, liver, blood, lung, cervical, prostate, breast, thyroid, skin, head, and neck [152,153,154]. Because inflammatory molecules modulate the physiological and pathological states of cancer and its surrounding microenvironment, and tumor initiation is said to occur as a result of prolonged exposure to inflammatory conditions, inhibition of inflammatory molecules could be a promising approach to managing cancer [155]. The NF-κB pathway and its associated molecules are key regulators of cancer cell survival and proliferation through increased expression of cell cycle related VEGF, inflammatory cytokines, and metastatic genes such as COX-2 [152]. Apigenin reduced prostate tumor volumes in mouse models through suppression of NF-κB activation [156]. In non-small cell lung cancer cell line A549, apigenin blocks the nuclear translocation of NF-κB, thereby suppressing the expression of tumorigenic genes such as Bcl-2, Mcl-1, and Bcl-xL. Apigenin also inhibits several signaling pathways including NF-κB and MAPK, inducing anti-cancer effects in malignant mesothelioma [157].

Macrophages are the most abundant innate immune cells in the tumor microenvironment that contribute to chronic low-grade inflammation, leading to tumor growth and metastasis through tumor neovascularization and matrix remodeling [158]. Apigenin induced apoptosis in mouse ANA-1 macrophage cell line through regulation of MAPK pathway and suppression of anti-apoptotic gene Bcl-2 [159]. Exposure to ultraviolet B (UVB) radiation results in acute inflammation due to production of various cytokines and chemokines via COX-2 expression and the resultant recruitment of neutrophils, monocytes, and macrophages, leading to acute responses such as skin edema or chronic inflammation, fibrosis, and cancer. Apigenin suppresses UVB-induced skin carcinogenesis through inhibition of inflammatory COX-2 and restoration of anti-angiogenic and anti-inflammatory thrombospondin-1 [160]. TNF-α contributes to breast cancer metastasis through the recruitment of tumor-infiltrating macrophages, neutrophils, and T cells, leading to immune evasion, tumor growth, and metastasis. Apigenin was shown to down-modulate TNF-α mediated upregulation of chemotactic protein, CCL2, granulocyte macrophage colony-stimulating factor (GM-CSF), IL-1α, and IL-6 in human triple-negative cells (MDA-MB-231 cells) [161].

**Figure 3:**
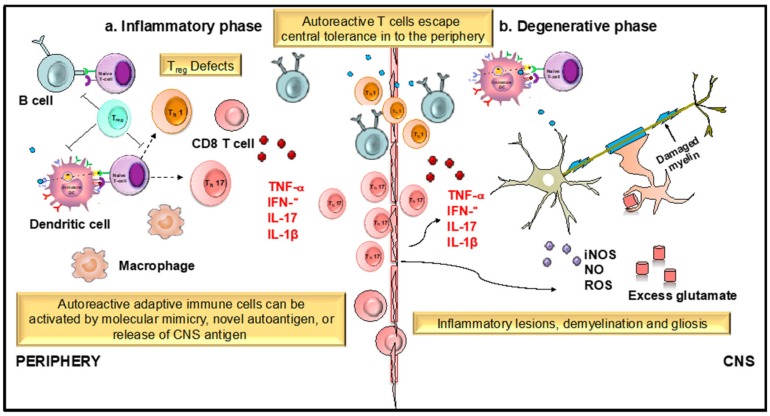
Role of dendritic cells (DCs) and T cells in the development and progression of multiple sclerosis (MS). MS is an immune mediated disease characterized by an initial inflammatory event consisting of presentation of as yet unknown antigens to CD8 T cells, their entry across the blood–brain barrier (BBB) into the central nervous system (CNS), and their subsequent reactivation by CNS resident DCs and microglial cells. This results in an inflammatory cascade involving secretion of several proinflammatory mediators such as cytokines IL-1β, IL-17, and TNF- α. The release of these cytokines initiates the degenerative phase that is characterized by increase in iNOS, NO, glutamate, and ROS, which brings about formation of inflammatory lesions, gliosis, and demyelination, which are the hallmarks of MS.

Apigenin, found abundantly in a variety of plants, herbs, and spices [136,162], has been utilized for centuries to treat diseases such as asthma, insomnia, Parkinson’s, neuralgia, and shingles [162,163], suggesting its potential use for both peripheral and CNS disorders. Apigenin has been shown to exert its neuroprotective effect via suppressing the expression of an inducible form of nitric oxide synthase (iNOS) and nitric oxide (NO) in microglial cells and macrophages [131]. Also, through regulation of adhesion molecules such as VCAM-1, ICAM-1, and E-selectin [164], which play a critical role in controlling leukocyte migration across the endothelial cells of BBB, apigenin might inhibit immune cells’ entry into the CNS and prevent neuroinflammation. However, it remains elusive whether apigenin or other flavones could serve as a potential treatment for neuroinflammatory disorders like multiple sclerosis, which affects approximately 400,000 people in the United States alone. Very little is known about the neuroprotective effects of apigenin and its related mechanism of action. In order to assess the therapeutic potential of apigenin in regulating neuroinflammation, we tested its efficacy in EAE models of relapse-remitting MS wherein apigenin reduced disease severity through inhibiting immune cell infiltration into the CNS and subsequent reduction in demyelination [132]. MS is an autoimmune disease with an as yet unknown etiologic agent mediated by an immunogenic response of auto-inflammatory T cells against the myelin sheath protecting the neurons. Dysregulation of DC function in MS can result from several possible reasons, which include, but are not limited to T-cell anergy in response to persistent antigens displayed by long-lived lymphoid DCs and functional abnormality of DCs (Figure 3). The infiltration of DCs from the periphery during neuroinflammatory autoimmunity has been studied extensively, particularly in EAE models of MS wherein DCs infiltrating from the blood increase with the increasing clinical severity of EAE. In fact, evidence shows that they interact with naive CD4^+^ T cells, driving T_h_17 differentiation, a T cell subset involved in chronic inflammatory disease [165,166]. Hence, the regulation of DC functions and its transmigration into the CNS holds the key to prevent the detrimental effects of immune infiltration in MS. Current MS therapies such as Natalizumab and dimethyl fumarate (DMF) that regulate leucocyte entry into the CNS have shown potential in controlling symptoms and relapse [167]. However, most of these do not control the progressive form of the MS and are often associated with significant side effects, emphasizing the need for and value of identifying safer, alternate therapies that could provide clinical level benefits for the debilitating diseases of the CNS.

### 3.2. Apigenin Mediated Modulation in Dendritic Cell Phenotypical and Functional Maturation

**Figure 4:**
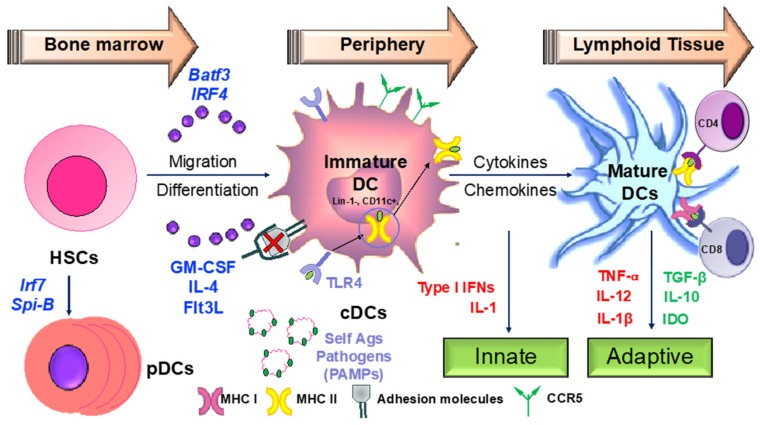
Dendritic cells as sentinels of the immune system. DCs orchestrate the immune response initiating both the innate and adaptive branches of the immune system. Any dysregulation in their activity is the key to development of chronic inflammatory and autoimmune conditions. IFN—interferon; GM-CSF—granulocyte macrophage colony-stimulating factor; HSC—hematopoietic stem cell(s); MHC—major histocompatibility complex; TLR—toll-like receptor.

Hematopoeitic stem cells in the bone marrow differentiate into plasmacytoid DCs from lymphoid progenitors in the presence of transcription factors such as like Irf7 and Spi-B [168] (Figure 4). DC progenitors in the bone marrow also give rise to circulating precursors in the presence of other factors like Batf3 and Irf4 that home to tissues, where they reside as immature cells with high phagocytic capacity [169]. Following tissue damage, immature DCs capture antigens through PRRs such as TLRs, and initiate the innate response through the secretion of IL-1 and type I interferons. Following antigen capture, immature DCs also subsequently migrate to the lymphoid organs, where they select rare antigen-specific T cells, thereby initiating adaptive immune responses. T cells are also educated by DCs to recognize and tolerate self-antigens. Sensing of microbial stimuli through PRRs causes DCs to enter the process of maturation, which involves the upregulation of major histocompatibility complex (MHC) molecules and co-stimulatory molecules. Peptide-loaded MHC molecules are recognized by Ag-specific T cells via the T-cell receptor (TCR), constituting signal 1 of T cell activation. Signal 2 consists of binding of costimulatory molecules on DCs to CD28/CD40L on T cells. Activated T cells in turn help DCs in terminal maturation through the ligation of CD40 and CD80/86. The final step in T cell activation is signal 3; the release of inflammatory cytokines and chemokines promoting the differentiation of naïve antigen-specific T cells into effector cells, as well as the activation of various other types of immune cells by the dendritic cells. Therapeutic agents targeting the various steps involved in DC-mediated T cell activation may be critical in the amelioration of various chronic inflammatory diseases.

Various flavonoids, described earlier in this review, inhibit the inflammatory functions of DCs. The role of apigenin on DC maturation and function has been investigated in murine bone marrow derived DCs, where inhibition of p65 translocation has been linked to downmodulation in cell surface expression of DC co-stimulatory molecules and antigen capture [170]. Apigenin reduced the severity of arthritis in a collagen-induced arthritis mouse model by reducing proinflammatory cytokine secretion from serum and supernatants from lymph node DCs. DCs from the apigenin-treated mice also exhibited low expression of MHC and co-stimulatory molecules [171]. More recently, apigenin was seen to reduce the expression of co-stimulatory CD80, CD86, and MHC II on murine splenic CD11c+ DCs. Additionally, LPS-matured splenic DCs pulsed with ovalbumin (OVA)323−339 and treated with apigenin impaired OVA-specific T cell proliferation [172]. However, the molecular players involved in the apigenin mediated control of DC function are still mostly unknown. It is also unclear whether apigenin is able to modulate DC phenotype and functional characteristics to regulate antigen-specific T cells in neuroinflammatory conditions. Hence, we investigated the effects of apigenin in EAE mice and reported disease attenuation and reduced demyelination. Amelioration in the disease phenotype was dictated by reduced CNS infiltration of myeloid immune cells. Functionality of both T_h_1 and T_h_17 cells was impaired and FoxP3+ T_reg_ cell numbers were seen to be boosted in apigenin-treated EAE mice. To evaluate whether these protective effects of apigenin are mediated by changes in DC phenotype and function, we investigated the effects of apigenin on human peripheral blood DCs. Unpublished data from these studies suggest that apigenin reduced cell surface expression of key antigen presentation and co-stimulatory markers and reduced the secretion of proinflammatory cytokines in LPS-matured DCs treated with apigenin in a RelB-dependent manner. It is known that NF-κB activation is required for T-cell activation by DCs, primarily through the canonical NF-κB pathway [173]. NF-κB consists of a family of five Rel proteins; namely, c-Rel, RelA/p65, RelB, NF-κB1 (p50 and its precursor, p105), and NF-κB2 (p52 and its precursor, p100), of which p65 and p50 predominantly compose the canonical pathway. Recent findings have increasingly suggested a role of NF-κB protein RelB in DC maturation, their antigen presenting functions, and DC-mediated immunity [173,174]. In mature DCs, RelB is upregulated and translocated into the nuclei in response to various maturation signals [175]. Additionally, the apigenin-induced changes in blood DCs lead to T-cell polarization away from T_h_1 and T_h_17 cells towards T_reg_ cells, as was seen in the EAE mice treated with apigenin. Thus, a DC-central anti-inflammatory agent could be key in resolving CNS inflammation and the resultant pathologies in various neurodegenerative diseases.

## 4. Development of Apigenin as a Viable Candidate for Anti-Neuroinflammatory Treatment

As predicted by the World Economic Forum, within the next 16 years, management of chronic disease including neuroinflammation is predicted to cost the world a staggering $47 trillion in treatment and lost wages. The treatments currently available are rarely curative and have serious side effects. The use of plant-based substances for the treatment of various mental ailments has been prevalent for centuries [176]. Of these, flavonoids are an important group of more than 4000 polyphenolic compounds possessing a common phenylbenzopyrone structure (C6-C3-C6), which allows a wide range of biological activities [177,178]. Among other related flavonoids, apigenin, a naturally occurring plant flavone, is found in abundance in common fruits and vegetables such as parsley, tea, chamomile, wheat sprouts, and some seasonings. It represents about 0.8% of the total flavonoids consumed on a daily basis by the U.S. population, estimated by the department of food science and human nutrition [179]. For the successful development of any natural product lead compound as a therapeutic entity, several important characteristics such as increased bioavailability, long half-life, and slow absorption and excretion need to be fulfilled. Additionally, the bioactive molecule should be able to convene at the target organ at an effective concentration unaltered, for it to exert a suitable biological effect. The bioavailability of a number of flavonoids has been extensively studied and the results have shown rapid excretion and extensive metabolism following ingestion. Most of the flavonoids are detected in the blood stream within a few minutes to a few hours, with half-lives not exceeding 48 h, after which they are excreted from the body. However, apigenin shows comparatively slower absorption kinetics and delayed plasma clearance in pathogen-free Wistar rats after single oral administration [180]. Apigenin is seen in the blood stream only 24 h after ingestion, with a relatively longer half-life of 91.8 h. Apigenin is also recovered at basal levels up to 10 days post ingestion, indicating slow excretion kinetics due to slow decomposition in the liver. These remarkable pharmacokinetics warrant further studies in human subjects to ascertain bioactive concentrations of apigenin required to obtain the anti-inflammatory pharmacological effects that have been reported in vitro.

Apigenin is a low molecular weight flavone (molecular weight = 270.24kDa), practically insoluble in water, partially soluble in hot alcohols, and completely soluble in potassium hydroxide (KOH) and dimethylsulfoxide (DMSO). Apigenin is a major constituent of chamomile and when prepared as chamomile tea, it has been used for centuries as a natural remedy for relieving indigestion and gastritis. Chamomile preparations have also been traditionally used in skin care products for the treatment of cutaneous inflammation and other skin disorders [138]. All flavonoids are synthesized in plants via a common pathway known as the shikimic acid pathway, which converts carbohydrate precursors to aromatic amino acids. Apigenin is synthesized through this pathway from its precursor Naringenin by the action of flavone synthase. Further, apigenin derivatives are also produced by O- or C-glycosylation, methylation, and hydroxylation of apigenin (Table 2). As mentioned earlier, it is relatively unclear whether naturally occurring structural modifications to the basic flavonoid enhances or suppresses its biological activities, especially its anti-inflammatory action. However, synthetic derivatives of apigenin have been reportedly generated to enhance various characteristics of the parent compound. The addition of different amines to the apigenin ring at the C-7 position generated two series of apigenin derivatives with enhanced anti-proliferative functions tested against different human cancer cell lines [181]. Apigenin amino acid prodrugs formed through synthesizing several amino acid conjugates including di- and tri-peptide analogs may improve solubility, enhancing its biological activity and usage. Several groups have synthesized and tested amino acid derivatives to improve the oral bioavailability of the plant flavone, tricin. The tricin-alanine-glutamic acid conjugate (T-Ala-Glu) exhibited excellent bioavailability after oral administration [182]. Similar derivatives can be generated with enhanced anti-inflammatory properties, as well as improving apigenin penetration across the BBB for its therapeutic potential in neuroinflammatory diseases.

Although the bioavailability and BBB penetration of apigenin is better than many other structurally related flavonoids, it may still not reach therapeutic levels to achieve the desired anti-inflammatory effects both in periphery and the CNS. Several research groups have devised different delivery systems to increase apigenin bioavailability. Nanosized drug delivery systems (NDDS) using liposome and polymer-based capsules for biocompatible delivery of large quantities of apigenin were designed and tested on human macrovascular endothelial cell line EAhy926 [183]. The NDDS showed optimal drug loading and good stability over extended time periods, and were non-toxic to the EAhy926 cells. With extended release characteristics, these NDDS can serve as nanocarriers for apigenin delivery to targeted organs to curb localized inflammation. The daily intake of apigenin is estimated to vary from 3.4 to 26 mg/day across the different countries and its consumption is largely based on lifestyle choices. To achieve therapeutic effects, apigenin will need to be ingested in its purified form with optimal release kinetics. Enteric polymer coated spheres were loaded with apigenin powder dispersed in an aqueous solution to allow targeted delivery to the intestine and colon, which are the main sites for absorption [184]. These pellets were able to ensure apigenin release within 1 h and achieved therapeutic anti-oxidant effects, making it an optimal delivery system for enhanced apigenin uptake. The BBB functions to not only allow the passage of essential nutrients and factors for the functioning of the brain, but also to limit the entry of therapeutic agents targeted against CNS abnormalities. This limits the number of drug entities that are able to cross the BBB to achieve the desired therapeutic effect. Though apigenin is a relatively small molecule that has been shown to cross the BBB, the intranasal delivery of apigenin, which bypasses the BBB and can gain entry in to the cerebrospinal fluid compartment via the olfactory pathway [185], could be explored to achieve therapeutic dosages of apigenin delivered directly to the CNS. An inhalable formulation of albumin nanoparticles loaded with apigenin has been constructed for the delivery of apigenin to lung tissue [186]. Effective anti-oxidant properties against lung injury were reported with this delivery system, making it a viable inhalable drug formulation. However, further testing in an in vivo model of neuroinflammation will provide useful information regarding CNS bioavailability and therapeutic reach.

## 5. Conclusions

A large body of epidemiological, in vitro, and in vivo studies have documented the anti-inflammatory properties of a wide variety of flavonoids in various chronic inflammatory conditions such as autoimmune diseases, cancer, diabetes, cardiovascular disorders, and neurodegenerative diseases. The beneficial biological activities of flavonoids are undoubtedly related to their structural composition and attributes, making them ideal lead candidates for the development of pharmaceuticals. A plethora of inflammatory molecules such as TNF-α, IL-1, IL-6, IL-17, and IFN-γ, which are secreted through the activation of several signaling pathways, predominantly the NF-κB pathway, has been shown to be suppressed upon treatment with different subclasses of flavonoids. Owing to the common phenol core, apigenin, a flavone, one of the most consumed flavonoids as part of the daily diet, also imparts health benefits such as anti-oxidant, anti-cancer, anti-inflammatory, and anti-viral properties. It is comparatively less toxic and non-mutagenic than other flavonoids of the same subclass, making it a better choice for development as a therapeutic entity. However, several in vivo studies in suitable animal models of chronic inflammation are warranted to understand its mode of action, its binding partners if any, effective dosages, and safe levels of administration. These studies will then pave the way for a large number of randomized clinical trials that will generate crucial data regarding its effectiveness as a stand-alone or combinatorial therapy in treatment of the different chronic inflammatory conditions such as MS. Because chronic diseases and their treatment leave debilitating and devastating effects on the patients, there is an urgent need to develop safer, more natural therapeutic agents for their management and cure. One of the most significant drawbacks of a natural product based drug compound is its low bioavailability. Though apigenin has been reported to possess remarkable pharmacokinetics, further development in terms of enhancing certain specific structural and chemical properties will be required to obtain more potent anti-inflammatory responses. Further, for its development as a viable treatment option against CNS neurodegenerative diseases, pre-clinical and clinical studies designed to investigate various drug formulations and deliver routes will also have to be carried out. Accumulating evidence so far suggests a very important role for apigenin in the treatment of various inflammatory disorders and bills it as a candidate worthy of in-depth investigation.

## Figures and Tables

**Table 1:** Subclasses of flavonoids. ERK—extracellular signal-regulated kinases, NF-κB—nuclear factor-kappa B; MAPK—mitogen-activated protein kinase; ROS—reactive oxygen species; COX-2—cyclooxygenase-2; IL—interleukin; TNF—tumor necrosis factor; iNOS—inducible NO synthase; PKC—protein kinase C, MDA—malondialdehyde , MMP—matrix metalloproteinase, FAK—focal adhesion kinase.

Class of Flavonoids	Chemical Structure	Dietary Source	Compound	Molecular Targets	Biological Function	Reference
Flavanol	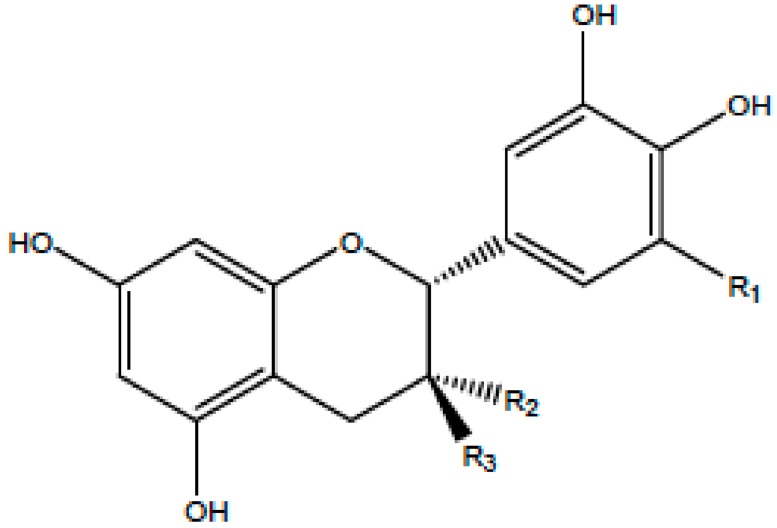	Tea, red wine, red grapes	Catechin, Epigallocatechin	↓ ERK, NF-κB, Rac1, AP-1, p38	Anti-carcinogenic	[2,9]
Flavone	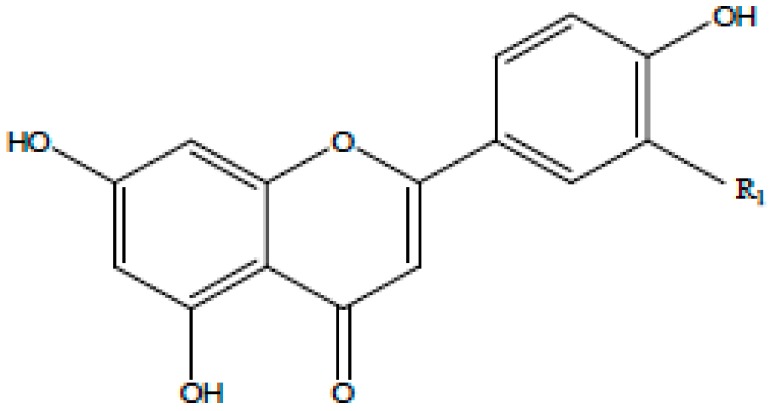	Fruit skins, red pepper, and tomato skin	Apigenin, Chrysin, and Luteolin	↓ Akt, ERK, caspase-12, caspase-3, MAPK, ROS, COX-2, IL-6, TNF-α, IL-1 β , iNOS, PGE2	Anti-inflammatory, anti-carcinogenic, neuroprotective	[10,11,12,13]
Flavonol	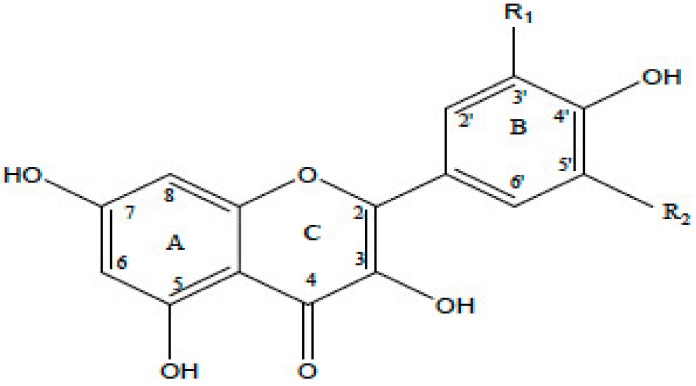	Onion, red wine, olive oil, berries, and grapefruit	Quercetin, Kaempferol, Myricetin, and Fisetin	↓ PKC, AP-1, H_2_O_2_, iNOS, MDA, citrate synthase, MMP-9,MMP-2, COX-2,ERK	Antioxidant, anti-inflammatory, neuroprotective reduce risk of vascular disease	[2,14]
Flavanone	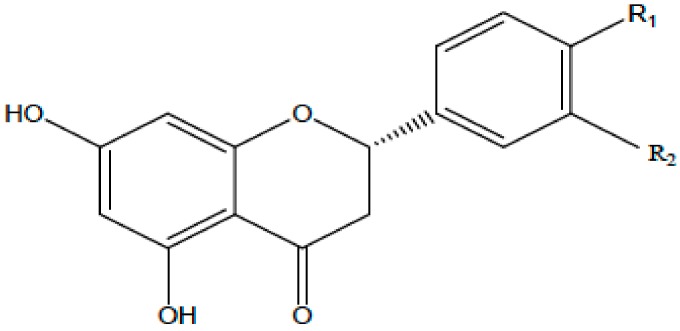	Citrus fruits, grapefruits, lemons, and oranges	Hesperetin, Naringenin	↓ROS, glutathione reductase, iNOS, 3-nitropropionic acid, COX2, NF-κB, IL-1β, TNF-α	Blood lipid-lowering and cholesterol-lowering agents, antiviral, antioxidant	[14]
Isoflavone	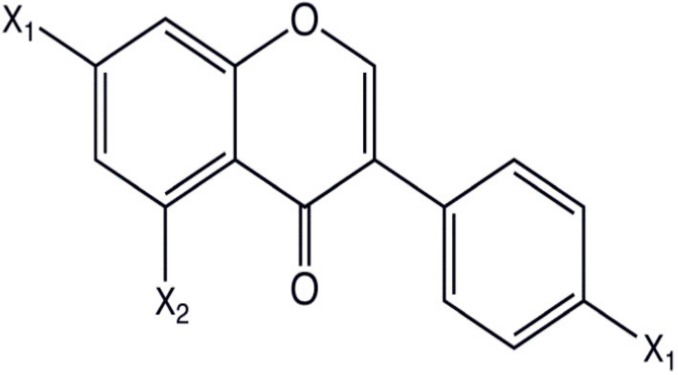	Soyabean	Genistin, Daidzin	↓ FAK, MAPK, NF-κB, AP-1, MMP-9, MMP-2	Anti-inflammatory, anti-cancer	
Anthocyanidin	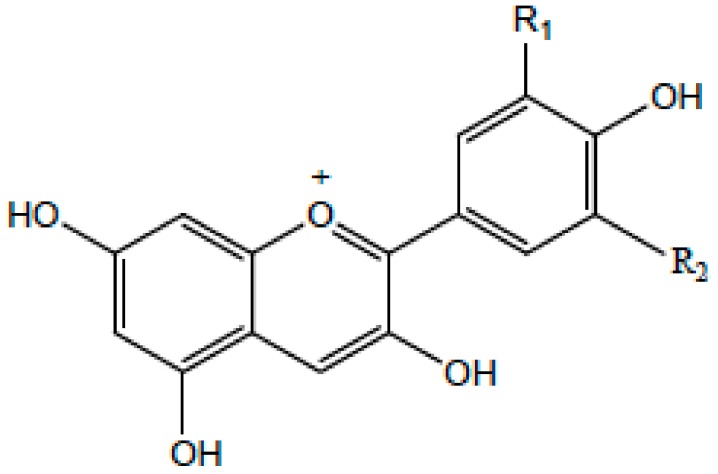	Cherry, Elsberry, and strawberry	Apigenidin, Cyanidin	↓MMP-9, MMP-2, ERK, AP-1, NFKB, MAPK,	Anti-inflammatory, antioxidant, anticancer, cardioprotective	
Flavanonol	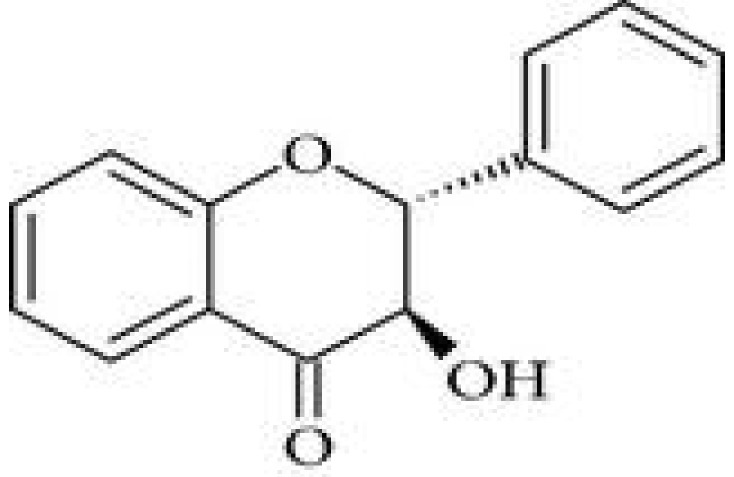	Limon, aurantium, Milk thistle	Taxifolin, Silibinin	↓ H_2_O_2_, iNOS, COX-2, IL-1β, TNF-α, NF-κB, IL-8, ROS	antioxidant, anti-inflammatory, neuroprotective, antiallergic, antitumor	

**Table 2:** Naturally occurring apigenin derivatives.

Name	Structure	Source	Modification	Biological Activity	Reference
Apiin	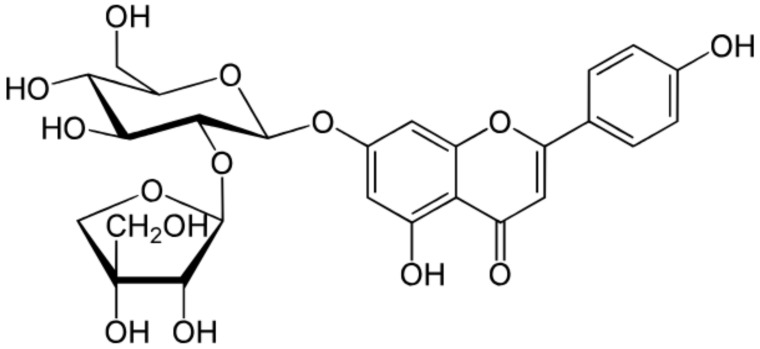	Parsley, Celery	Glycosylation, Hydroxylation	Anti-oxidant	[54,55]
Apigetrin	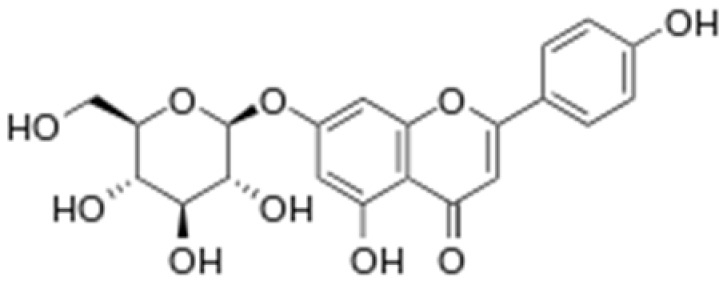	Roots of dandelion coffee	Glycosylation	Anti-inflammatory, anti-cancer	[55,56]
Vitexin	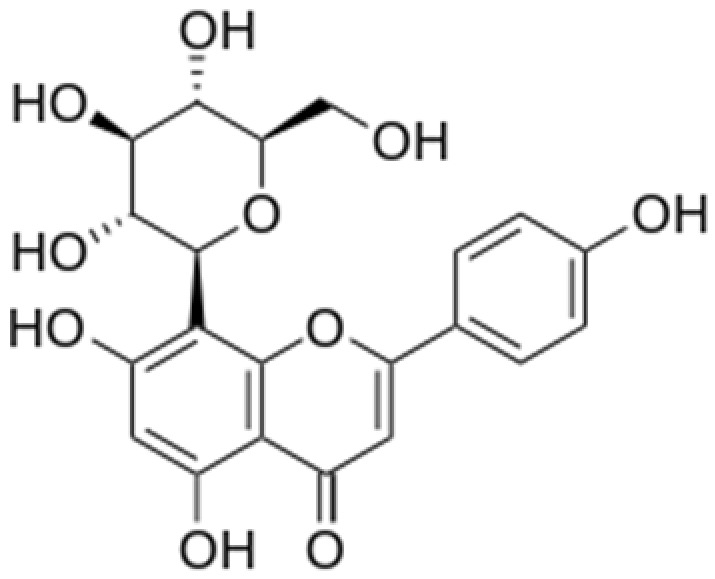	Mung bean, Bamboo leaves	Glycosylation	Anti-oxidant, neuroprotective, Anti-inflammatory	[55,57]
Isovitexin	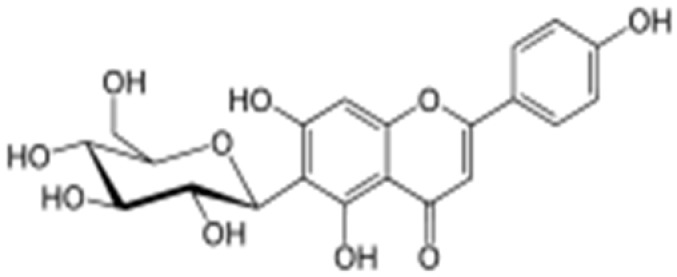	Mung bean, Ficus deltoidea	Glycosylation, Hydroxylation	Anti-inflammatory, anti-Alzheimer’s	[55,57]
Rhoifolin	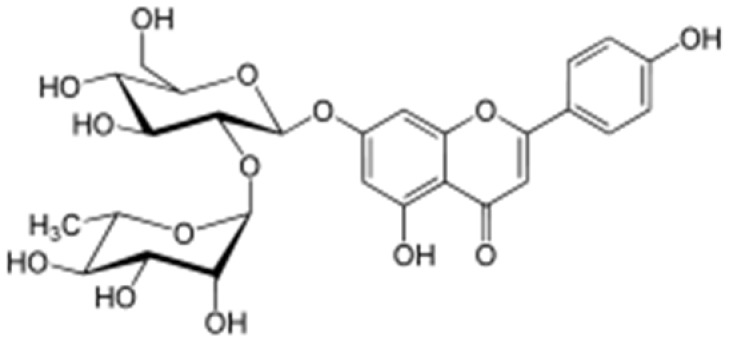	Orange,lupinus, Citrus grandis	Hydroxylation	Anti-microbial, anti-cancer, anti-inflammatory	[55,58]
Schaftoside	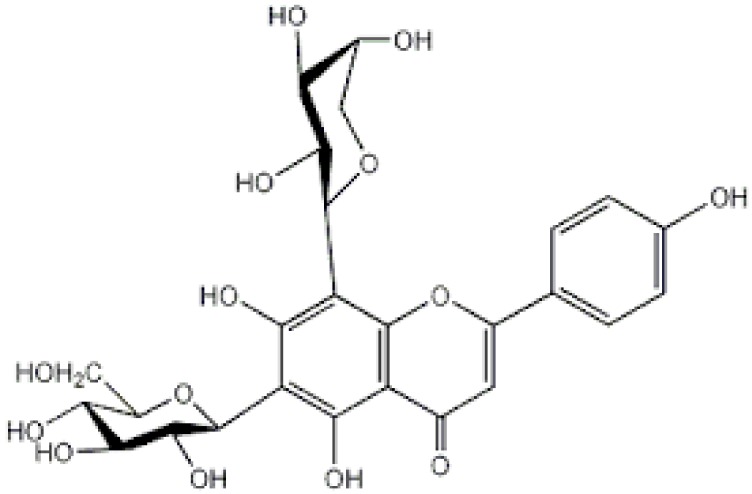	Arisaema heterophyllum	Glycosylation	Anti-melanogenic	[55,59]
Acacetin	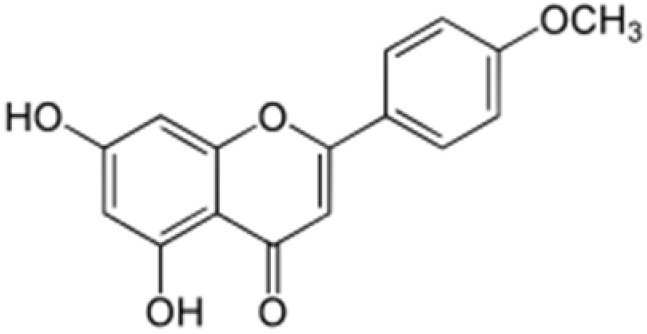	Turnera diffusa, Chrysanthemum morifolium	Methylation	Anti-inflammatory, antinociceptive	[55,60]
Genkwanin	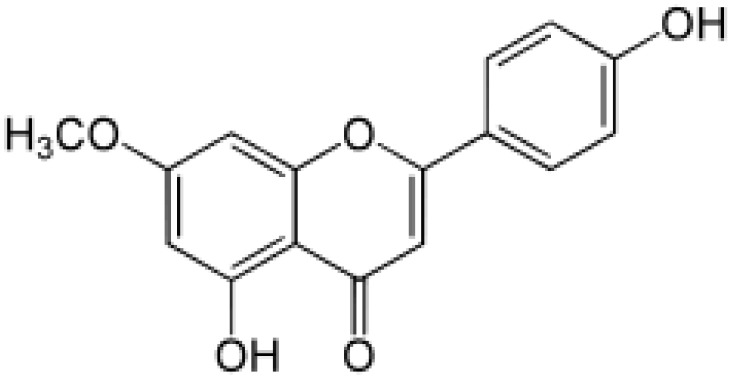	Genkwa flos, rosemary, seeds of Alnus glutinosa.	Methylation	Anti-tumor, anti-inflammatory	

## References

[1] Hunter P. (2012). The inflammation theory of disease. The growing realization that chronic inflammation is crucial in many diseases opens new avenues for treatment. EMBO Rep..

[2] Netea M.G., Balkwill F., Chonchol M., Cominelli F., Donath M.Y., Giamarellos-Bourboulis E.J., Golenbock D., Gresnigt M.S., Heneka M.T., Hoffman H.M. (2017). A guiding map for inflammation. Nat. Immunol..

[3] Ma K.C., Schenck E.J., Pabon M.A., Choi A.M.K. (2018). The Role of Danger Signals in the Pathogenesis and Perpetuation of Critical Illness. Am. J. Respir. Crit. Care Med..

[4] Pahwa R., Jialal I. (2018). Chronic Inflammation.

[5] Herwald H., Egesten A. (2016). On PAMPs and DAMPs. J. Innate Immun..

[6] Afonina I.S., Zhong Z., Karin M., Beyaert R. (2017). Limiting inflammation-the negative regulation of NF-kappaB and the NLRP3 inflammasome. Nat. Immunol..

[7] Gandhi J., Khera L., Gaur N., Paul C., Kaul R. (2017). Role of Modulator of Inflammation Cyclooxygenase-2 in Gammaherpesvirus Mediated Tumorigenesis. Front. Microbiol..

[8] Bleich S.N., Sherrod C., Chiang A., Boyd C., Wolff J., DuGoff E., Salzberg C., Anderson K., Leff B., Anderson G. (2015). Systematic Review of Programs Treating High-Need and High-Cost People with Multiple Chronic Diseases or Disabilities in the United States, 2008–2014. Prev. Chronic Dis..

[9] Nguyen N.H., Khera R., Ohno-Machado L., Sandborn W.J., Singh S. (2018). Annual Burden and Costs of Hospitalization for High-Need, High-Cost Patients with Chronic Gastrointestinal and Liver Diseases. Clin. Gastroenterol. Hepatol..

[10] Li P., Zheng Y., Chen X. (2017). Drugs for Autoimmune Inflammatory Diseases: From Small Molecule Compounds to Anti-TNF Biologics. Front. Pharmacol..

[11] Rengasamy K.R.R., Khan H., Gowrishankar S., Lagoa R.J.L., Mahomoodally F.M., Khan Z., Suroowan S., Tewari D., Zengin G., Hassan S.T.S. (2018). The role of flavonoids in autoimmune diseases: Therapeutic updates. Pharmacol. Ther..

[12] Baker D., Marta M., Pryce G., Giovannoni G., Schmierer K. (2017). Memory B Cells are Major Targets for Effective Immunotherapy in Relapsing Multiple Sclerosis. EBioMedicine.

[13] Felten R., Scher F., Sibilia J., Chasset F., Arnaud L. (2018). Advances in the treatment of systemic lupus erythematosus: From back to the future, to the future and beyond. Joint Bone Spine.

[14] Mak P., Leung Y.K., Tang W.Y., Harwood C., Ho S.M. (2006). Apigenin suppresses cancer cell growth through ERbeta. Neoplasia.

[15] Bernardini S., Tiezzi A., Laghezza Masci V., Ovidi E. (2018). Natural products for human health: An historical overview of the drug discovery approaches. Nat. Prod. Res..

[16] Dias D.A., Urban S., Roessner U. (2012). A historical overview of natural products in drug discovery. Metabolites.

[17] Thomford N.E., Senthebane D.A., Rowe A., Munro D., Seele P., Maroyi A., Dzobo K. (2018). Natural Products for Drug Discovery in the 21st Century: Innovations for Novel Drug Discovery. Int. J. Mol. Sci..

[18] Patwardhan B., Vaidya A.D. (2010). Natural products drug discovery: Accelerating the clinical candidate development using reverse pharmacology approaches. Indian J. Exp. Biol..

[19] Muller H., Brackhagen O., Brunne R., Henkel T., Reichel F. (2000). Natural products in drug discovery. Ernst Schering Res. Found. Workshop.

[20] Molinari G. (2009). Natural products in drug discovery: Present status and perspectives. Adv. Exp. Med. Biol..

[21] Newman D.J., Cragg G.M. (2016). Natural Products as Sources of New Drugs from 1981 to 2014. J. Nat. Prod..

[22] Cragg G.M., Newman D.J. (2013). Natural products: A continuing source of novel drug leads. Biochim. Biophys. Acta.

[23] Harvey A.L., Edrada-Ebel R., Quinn R.J. (2015). The re-emergence of natural products for drug discovery in the genomics era. Nat. Rev. Drug Discov..

[24] Patridge E., Gareiss P., Kinch M.S., Hoyer D. (2016). An analysis of FDA-approved drugs: Natural products and their derivatives. Drug Discov. Today.

[25] Shen B. (2015). A New Golden Age of Natural Products Drug Discovery. Cell.

[26] Aswad M., Rayan M., Abu-Lafi S., Falah M., Raiyn J., Abdallah Z., Rayan A. (2018). Nature is the best source of anti-inflammatory drugs: Indexing natural products for their anti-inflammatory bioactivity. Inflamm. Res..

[27] Attiq A., Jalil J., Husain K., Ahmad W. (2018). Raging the War Against Inflammation with Natural Products. Front. Pharmacol..

[28] Azab A., Nassar A., Azab A.N. (2016). Anti-Inflammatory Activity of Natural Products. Molecules.

[29] Furst R., Zundorf I. (2014). Plant-derived anti-inflammatory compounds: Hopes and disappointments regarding the translation of preclinical knowledge into clinical progress. Mediators Inflamm..

[30] Schafer G., Kaschula C.H. (2014). The immunomodulation and anti-inflammatory effects of garlic organosulfur compounds in cancer chemoprevention. Anticancer Agents Med. Chem..

[31] Lee J.C., Hou M.F., Huang H.W., Chang F.R., Yeh C.C., Tang J.Y., Chang H.W. (2013). Marine algal natural products with anti-oxidative, anti-inflammatory, and anti-cancer properties. Cancer Cell. Int..

[32] Wei W.C., Sung P.J., Duh C.Y., Chen B.W., Sheu J.H., Yang N.S. (2013). Anti-inflammatory activities of natural products isolated from soft corals of Taiwan between 2008 and 2012. Mar. Drugs.

[33] Panche A.N., Diwan A.D., Chandra S.R. (2016). Flavonoids: An overview. J. Nutr. Sci..

[34] Patel K., Singh G.K., Patel D.K. (2018). A Review on Pharmacological and Analytical Aspects of Naringenin. Chin. J. Integr. Med..

[35] Tungmunnithum D., Thongboonyou A., Pholboon A., Yangsabai A. (2018). Flavonoids and Other Phenolic Compounds from Medicinal Plants for Pharmaceutical and Medical Aspects: An Overview. Medicines.

[36] Kumar S., Pandey A.K. (2013). Chemistry and biological activities of flavonoids: An overview. Sci. World J..

[37] Brodowska K.M. (2017). Natural flavonoids: Classification, potential role, and application of flavonoid analogues. Eur. J. Biol. Res..

[38] Lago J.H., Toledo-Arruda A.C., Mernak M., Barrosa K.H., Martins M.A., Tiberio I.F., Prado C.M. (2014). Structure-activity association of flavonoids in lung diseases. Molecules.

[39] Theoharides T.C., Alexandrakis M., Kempuraj D., Lytinas M. (2001). Anti-inflammatory actions of flavonoids and structural requirements for new design. Int. J. Immunopathol. Pharmacol..

[40] Kim H.P., Son K.H., Chang H.W., Kang S.S. (2004). Anti-inflammatory plant flavonoids and cellular action mechanisms. J. Pharmacol. Sci..

[41] Yao L.H., Jiang Y.M., Shi J., Tomas-Barberan F.A., Datta N., Singanusong R., Chen S.S. (2004). Flavonoids in food and their health benefits. Plant. Foods Hum. Nutr..

[42] Todoric J., Antonucci L., Karin M. (2016). Targeting Inflammation in Cancer Prevention and Therapy. Cancer Prev. Res..

[43] Ostrand-Rosenberg S., Sinha P. (2009). Myeloid-derived suppressor cells: Linking inflammation and cancer. J. Immunol..

[44] Yang L., Huang J., Ren X., Gorska A.E., Chytil A., Aakre M., Carbone D.P., Matrisian L.M., Richmond A., Lin P.C. (2008). Abrogation of TGF beta signaling in mammary carcinomas recruits Gr-1+CD11b+ myeloid cells that promote metastasis. Cancer Cell.

[45] Eiro N., Vizoso F.J. (2012). Inflammation and cancer. World J. Gastrointest. Surg..

[46] Akiyama T., Ishida J., Nakagawa S., Ogawara H., Watanabe S., Itoh N., Shibuya M., Fukami Y. (1987). Genistein, a specific inhibitor of tyrosine-specific protein kinases. J. Biol. Chem..

[47] Constantinou A.I., Kamath N., Murley J.S. (1998). Genistein inactivates bcl-2, delays the G2/M phase of the cell cycle, and induces apoptosis of human breast adenocarcinoma MCF-7 cells. Eur. J. Cancer.

[48] Fotsis T., Pepper M., Adlercreutz H., Fleischmann G., Hase T., Montesano R., Schweigerer L. (1993). Genistein, a dietary-derived inhibitor of in vitro angiogenesis. Proc. Natl. Acad. Sci. USA.

[49] Markovits J., Linassier C., Fosse P., Couprie J., Pierre J., Jacquemin-Sablon A., Saucier J.M., Le Pecq J.B., Larsen A.K. (1989). Inhibitory effects of the tyrosine kinase inhibitor genistein on mammalian DNA topoisomerase II. Cancer Res..

[50] Matsukawa Y., Marui N., Sakai T., Satomi Y., Yoshida M., Matsumoto K., Nishino H., Aoike A. (1993). Genistein arrests cell cycle progression at G2-M. Cancer Res..

[51] Chahar M.K., Sharma N., Dobhal M.P., Joshi Y.C. (2011). Flavonoids: A versatile source of anticancer drugs. Pharmacogn. Rev..

[52] Mojzis J., Varinska L., Mojzisova G., Kostova I., Mirossay L. (2008). Antiangiogenic effects of flavonoids and chalcones. Pharmacol. Res..

[53] Schmitz M.L., Kracht M. (2016). Cyclin-Dependent Kinases as Coregulators of Inflammatory Gene Expression. Trends Pharmacol. Sci..

[54] Pollack R.M., Donath M.Y., LeRoith D., Leibowitz G. (2016). Anti-inflammatory Agents in the Treatment of Diabetes and Its Vascular Complications. Diabetes Care.

[55] Garcia C., Feve B., Ferre P., Halimi S., Baizri H., Bordier L., Guiu G., Dupuy O., Bauduceau B., Mayaudon H. (2010). Diabetes and inflammation: Fundamental aspects and clinical implications. Diabetes Metab..

[56] Zhou R., Tardivel A., Thorens B., Choi I., Tschopp J. (2010). Thioredoxin-interacting protein links oxidative stress to inflammasome activation. Nat. Immunol..

[57] Vinayagam R., Xu B. (2015). Antidiabetic properties of dietary flavonoids: A cellular mechanism review. Nutr. Metab..

[58] Testa R., Bonfigli A.R., Genovese S., De Nigris V., Ceriello A. (2016). The Possible Role of Flavonoids in the Prevention of Diabetic Complications. Nutrients.

[59] Choi E.J., Kim G.H. (2009). 5-Fluorouracil combined with apigenin enhances anticancer activity through induction of apoptosis in human breast cancer MDA-MB-453 cells. Oncol. Rep..

[60] Abuohashish H.M., Al-Rejaie S.S., Al-Hosaini K.A., Parmar M.Y., Ahmed M.M. (2013). Alleviating effects of morin against experimentally-induced diabetic osteopenia. Diabetol. Metab. Syndr..

[61] Niture N.T., Ansari A.A., Naik S.R. (2014). Anti-hyperglycemic activity of rutin in streptozotocin-induced diabetic rats: An effect mediated through cytokines, antioxidants and lipid biomarkers. Indian J. Exp. Biol..

[62] Sirovina D., Orsolic N., Koncic M.Z., Kovacevic G., Benkovic V., Gregorovic G. (2013). Quercetin vs chrysin: Effect on liver histopathology in diabetic mice. Hum. Exp. Toxicol..

[63] Visnagri A., Kandhare A.D., Chakravarty S., Ghosh P., Bodhankar S.L. (2014). Hesperidin, a flavanoglycone attenuates experimental diabetic neuropathy via modulation of cellular and biochemical marker to improve nerve functions. Pharm. Biol..

[64] Jadhav R., Puchchakayala G. (2012). Hypoglycemic and Antidiabetic Activity of Flavonoids: Boswellic acid, ellagic acid, quercetin, rutin on streptozotocin-nicotinamide induced type 2 diabetic rats. Int. J. Pharmacy Pharm. Sci..

[65] Agrawal Y.O., Sharma P.K., Shrivastava B., Ojha S., Upadhya H.M., Arya D.S., Goyal S.N. (2014). Hesperidin produces cardioprotective activity via PPAR-gamma pathway in ischemic heart disease model in diabetic rats. PLoS ONE.

[66] Ahad A., Ganai A.A., Mujeeb M., Siddiqui W.A. (2014). Chrysin, an anti-inflammatory molecule, abrogates renal dysfunction in type 2 diabetic rats. Toxicol. Appl. Pharmacol..

[67] Li R., Zang A., Zhang L., Zhang H., Zhao L., Qi Z., Wang H. (2014). Chrysin ameliorates diabetes-associated cognitive deficits in Wistar rats. Neurol. Sci..

[68] Calle M.C., Fernandez M.L. (2012). Inflammation and type 2 diabetes. Diabetes Metab..

[69] Nelson V.L., Nguyen H.C.B., Garcia-Canaveras J.C., Briggs E.R., Ho W.Y., DiSpirito J.R., Marinis J.M., Hill D.A., Lazar M.A. (2018). PPARgamma is a nexus controlling alternative activation of macrophages via glutamine metabolism. Genes Dev..

[70] Saini V. (2010). Molecular mechanisms of insulin resistance in type 2 diabetes mellitus. World J. Diabetes.

[71] Ding L., Jin D., Chen X. (2010). Luteolin enhances insulin sensitivity via activation of PPARgamma transcriptional activity in adipocytes. J. Nutr. Biochem..

[72] Liu Y., Fu X., Lan N., Li S., Zhang J., Wang S., Li C., Shang Y., Huang T., Zhang L. (2014). Luteolin protects against high fat diet-induced cognitive deficits in obesity mice. Behav. Brain Res..

[73] Salaritabar A., Darvishi B., Hadjiakhoondi F., Manayi A., Sureda A., Nabavi S.F., Fitzpatrick L.R., Nabavi S.M., Bishayee A. (2017). Therapeutic potential of flavonoids in inflammatory bowel disease: A comprehensive review. World J. Gastroenterol..

[74] Matricon J., Barnich N., Ardid D. (2010). Immunopathogenesis of inflammatory bowel disease. Self Nonself.

[75] Vezza T., Rodriguez-Nogales A., Algieri F., Utrilla M.P., Rodriguez-Cabezas M.E., Galvez J. (2016). Flavonoids in Inflammatory Bowel Disease: A Review. Nutrients.

[76] Hart A.L., Al-Hassi H.O., Rigby R.J., Bell S.J., Emmanuel A.V., Knight S.C., Kamm M.A., Stagg A.J. (2005). Characteristics of intestinal dendritic cells in inflammatory bowel diseases. Gastroenterology.

[77] Niess J.H. (2008). Role of mucosal dendritic cells in inflammatory bowel disease. World J. Gastroenterol..

[78] Yoshida T. (2018). Concise Commentary: Quercetin Flavonoid of the Month or IBD Therapy?. Dig. Dis. Sci..

[79] Hoensch H.P., Weigmann B. (2018). Regulation of the intestinal immune system by flavonoids and its utility in chronic inflammatory bowel disease. World J. Gastroenterol..

[80] Schneider M.J., Abdel-Aziz H., Efferth T. (2014). Phytochemicals for the treatment of inflammatory bowel diseases. Phytochem. Rev..

[81] Hur S.J., Kang S.H., Jung H.S., Kim S.C., Jeon H.S., Kim I.H., Lee J.D. (2012). Review of natural products actions on cytokines in inflammatory bowel disease. Nutr. Res..

[82] Harald P., Hoensch R.O. (2015). The value of flavonoids for the human nutrition: Short review and perspectives. Clin. Nutr. Exp..

[83] Bian Y., Liu P., Zhong J., Hu Y., Zhuang S., Fan K., Liu Z. (2018). Quercetin Attenuates Adhesion Molecule Expression in Intestinal Microvascular Endothelial Cells by Modulating Multiple Pathways. Dig. Dis Sci..

[84] Comalada M., Camuesco D., Sierra S., Ballester I., Xaus J., Galvez J., Zarzuelo A. (2005). In vivo quercitrin anti-inflammatory effect involves release of quercetin, which inhibits inflammation through down-regulation of the NF-kappaB pathway. Eur. J. Immunol..

[85] Kwon K.H., Murakami A., Tanaka T., Ohigashi H. (2005). Dietary rutin, but not its aglycone quercetin, ameliorates dextran sulfate sodium-induced experimental colitis in mice: Attenuation of pro-inflammatory gene expression. Biochem. Pharmacol..

[86] Sanchez de Medina F., Galvez J., Romero J.A., Zarzuelo A. (1996). Effect of quercitrin on acute and chronic experimental colitis in the rat. J. Pharmacol. Exp. Ther..

[87] Zhang Y.S., Wang F., Cui S.X., Qu X.J. (2018). Natural dietary compound naringin prevents azoxymethane/dextran sodium sulfate-induced chronic colorectal inflammation and carcinogenesis in mice. Cancer Biol. Ther..

[88] Ahmed A., Wong R.J., Harrison S.A. (2015). Nonalcoholic Fatty Liver Disease Review: Diagnosis, Treatment, and Outcomes. Clin. Gastroenterol. Hepatol..

[89] Chao C.Y., Battat R., Al Khoury A., Restellini S., Sebastiani G., Bessissow T. (2016). Co-existence of non-alcoholic fatty liver disease and inflammatory bowel disease: A review article. World J. Gastroenterol..

[90] Fotbolcu H., Zorlu E. (2016). Nonalcoholic fatty liver disease as a multi-systemic disease. World J. Gastroenterol..

[91] Bibbo S., Ianiro G., Dore M.P., Simonelli C., Newton E.E., Cammarota G. (2018). Gut Microbiota as a Driver of Inflammation in Nonalcoholic Fatty Liver Disease. Mediators Inflamm..

[92] Duarte N., Coelho I.C., Patarrao R.S., Almeida J.I., Penha-Goncalves C., Macedo M.P. (2015). How Inflammation Impinges on NAFLD: A Role for Kupffer Cells. Biomed. Res. Int..

[93] Van De Wier B., Koek G.H., Bast A., Haenen G.R. (2017). The potential of flavonoids in the treatment of non-alcoholic fatty liver disease. Crit. Rev. Food Sci. Nutr..

[94] Haddad Y., Vallerand D., Brault A., Haddad P.S. (2011). Antioxidant and hepatoprotective effects of silibinin in a rat model of nonalcoholic steatohepatitis. Evid. Based Complement. Alternat. Med..

[95] Salamone F., Galvano F., Cappello F., Mangiameli A., Barbagallo I., Li Volti G. (2012). Silibinin modulates lipid homeostasis and inhibits nuclear factor kappa B activation in experimental nonalcoholic steatohepatitis. Transl. Res..

[96] Kim M., Yang S.G., Kim J.M., Lee J.W., Kim Y.S., Lee J.I. (2012). Silymarin suppresses hepatic stellate cell activation in a dietary rat model of non-alcoholic steatohepatitis: Analysis of isolated hepatic stellate cells. Int. J. Mol. Med..

[97] Yoo N.Y., Jeon S., Nam Y., Park Y.J., Won S.B., Kwon Y.H. (2015). Dietary Supplementation of Genistein Alleviates Liver Inflammation and Fibrosis Mediated by a Methionine-Choline-Deficient Diet in db/db Mice. J. Agric. Food Chem..

[98] Ji G., Yang Q., Hao J., Guo L., Chen X., Hu J., Leng L., Jiang Z. (2011). Anti-inflammatory effect of genistein on non-alcoholic steatohepatitis rats induced by high fat diet and its potential mechanisms. Int. Immunopharmacol..

[99] Andersen C., Schjoldager J.G., Tortzen C.G., Vegge A., Hufeldt M.R., Skaanild M.T., Vogensen F.K., Kristiansen K., Hansen A.K., Nielsen J. (2013). 2-heptyl-formononetin increases cholesterol and induces hepatic steatosis in mice. Biomed. Res. Int..

[100] Gao M., Ma Y., Liu D. (2013). Rutin suppresses palmitic acids-triggered inflammation in macrophages and blocks high fat diet-induced obesity and fatty liver in mice. Pharm. Res..

[101] Marcolin E., San-Miguel B., Vallejo D., Tieppo J., Marroni N., Gonzalez-Gallego J., Tunon M.J. (2012). Quercetin treatment ameliorates inflammation and fibrosis in mice with nonalcoholic steatohepatitis. J. Nutr..

[102] Dorn C., Kraus B., Motyl M., Weiss T.S., Gehrig M., Scholmerich J., Heilmann J., Hellerbrand C. (2010). Xanthohumol, a chalcon derived from hops, inhibits hepatic inflammation and fibrosis. Mol. Nutr. Food Res..

[103] Zhu W., Jia Q., Wang Y., Zhang Y., Xia M. (2012). The anthocyanin cyanidin-3-O-beta-glucoside, a flavonoid, increases hepatic glutathione synthesis and protects hepatocytes against reactive oxygen species during hyperglycemia: Involvement of a cAMP-PKA-dependent signaling pathway. Free Radic. Biol. Med..

[104] Ruparelia N., Chai J.T., Fisher E.A., Choudhury R.P. (2017). Inflammatory processes in cardiovascular disease: A route to targeted therapies. Nat. Rev. Cardiol..

[105] Golia E., Limongelli G., Natale F., Fimiani F., Maddaloni V., Pariggiano I., Bianchi R., Crisci M., D’Acierno L., Giordano R. (2014). Inflammation and cardiovascular disease: From pathogenesis to therapeutic target. Curr. Atheroscler. Rep..

[106] Kostyuk V.A., Potapovich A.I., Suhan T.O., de Luca C., Korkina L.G. (2011). Antioxidant and signal modulation properties of plant polyphenols in controlling vascular inflammation. Eur. J. Pharmacol..

[107] Al-Awwadi N.A., Araiz C., Bornet A., Delbosc S., Cristol J.P., Linck N., Azay J., Teissedre P.L., Cros G. (2005). Extracts enriched in different polyphenolic families normalize increased cardiac NADPH oxidase expression while having differential effects on insulin resistance, hypertension, and cardiac hypertrophy in high-fructose-fed rats. J. Agric. Food Chem..

[108] Boesch-Saadatmandi C., Loboda A., Wagner A.E., Stachurska A., Jozkowicz A., Dulak J., Doring F., Wolffram S., Rimbach G. (2011). Effect of quercetin and its metabolites isorhamnetin and quercetin-3-glucuronide on inflammatory gene expression: Role of miR-155. J. Nutr. Biochem..

[109] Osiecki H. (2004). The role of chronic inflammation in cardiovascular disease and its regulation by nutrients. Altern. Med. Rev..

[110] Droke E.A., Hager K.A., Lerner M.R., Lightfoot S.A., Stoecker B.J., Brackett D.J., Smith B.J. (2007). Soy isoflavones avert chronic inflammation-induced bone loss and vascular disease. J. Inflamm..

[111] Garcia-Lafuente A., Guillamon E., Villares A., Rostagno M.A., Martinez J.A. (2009). Flavonoids as anti-inflammatory agents: Implications in cancer and cardiovascular disease. Inflamm. Res..

[112] Onasanwo S.A., Velagapudi R., El-Bakoush A., Olajide O.A. (2016). Inhibition of neuroinflammation in BV2 microglia by the biflavonoid kolaviron is dependent on the Nrf2/ARE antioxidant protective mechanism. Mol. Cell. Biochem..

[113] Gonzalez H., Pacheco R. (2014). T-cell-mediated regulation of neuroinflammation involved in neurodegenerative diseases. J. Neuroinflamm..

[114] Ota A., Ulrih N.P. (2017). An Overview of Herbal Products and Secondary Metabolites Used for Management of Type Two Diabetes. Front. Pharmacol..

[115] Manuel S.L., Rahman S., Wigdahl B., Khan Z.K., Jain P. (2007). Dendritic cells in autoimmune diseases and neuroinflammatory disorders. Front. Biosci..

[116] Wu G.F., Laufer T.M. (2007). The role of dendritic cells in multiple sclerosis. Curr. Neurol. Neurosci. Rep..

[117] Steinman R.M., Cohn Z.A. (1973). Identification of a novel cell type in peripheral lymphoid organs of mice. I. Morphology, quantitation, tissue distribution. J. Exp. Med..

[118] Sagar D., Foss C., El Baz R., Pomper M.G., Khan Z.K., Jain P. (2012). Mechanisms of dendritic cell trafficking across the blood-brain barrier. J. Neuroimmune Pharmacol..

[119] Sagar D., Lamontagne A., Foss C.A., Khan Z.K., Pomper M.G., Jain P. (2012). Dendritic cell CNS recruitment correlates with disease severity in EAE via CCL2 chemotaxis at the blood-brain barrier through paracellular transmigration and ERK activation. J. Neuroinflamm..

[120] Pashenkov M., Huang Y.M., Kostulas V., Haglund M., Soderstrom M., Link H. (2001). Two subsets of dendritic cells are present in human cerebrospinal fluid. Brain.

[121] Greter M., Heppner F.L., Lemos M.P., Odermatt B.M., Goebels N., Laufer T., Noelle R.J., Becher B. (2005). Dendritic cells permit immune invasion of the CNS in an animal model of multiple sclerosis. Nat. Med..

[122] Li K.C., Ho Y.L., Hsieh W.T., Huang S.S., Chang Y.S., Huang G.J. (2015). Apigenin-7-glycoside prevents LPS-induced acute lung injury via downregulation of oxidative enzyme expression and protein activation through inhibition of MAPK phosphorylation. Int. J. Mol. Sci..

[123] Vauzour D., Vafeiadou K., Rodriguez-Mateos A., Rendeiro C., Spencer J.P. (2008). The neuroprotective potential of flavonoids: A multiplicity of effects. Genes Nutr..

[124] Suk K. (2007). Research Focus on Natural Products and the Body’s Immune and Inflammatory Systems.

[125] Farooqui A.A. (2016). Therapeutic Potentials of Curcumin for Alzheimer Disease.

[126] Sternberg Z., Chadha K., Lieberman A., Drake A., Hojnacki D., Weinstock-Guttman B., Munschauer F. (2009). Immunomodulatory responses of peripheral blood mononuclear cells from multiple sclerosis patients upon in vitro incubation with the flavonoid luteolin: Additive effects of IFN-beta. J. Neuroinflamm..

[127] Kim J.S., Jobin C. (2005). The flavonoid luteolin prevents lipopolysaccharide-induced NF-kappaB signalling and gene expression by blocking IkappaB kinase activity in intestinal epithelial cells and bone-marrow derived dendritic cells. Immunology.

[128] Kim Y.J., Choi S.E., Lee M.W., Lee C.S. (2008). Taxifolin glycoside inhibits dendritic cell responses stimulated by lipopolysaccharide and lipoteichoic acid. J. Pharm. Pharmacol..

[129] Lee J.S., Kim S.G., Kim H.K., Lee T.H., Jeong Y.I., Lee C.M., Yoon M.S., Na Y.J., Suh D.S., Park N.C. (2007). Silibinin polarizes Th1/Th2 immune responses through the inhibition of immunostimulatory function of dendritic cells. J. Cell. Physiol..

[130] Yoneyama S., Kawai K., Tsuno N.H., Okaji Y., Asakage M., Tsuchiya T., Yamada J., Sunami E., Osada T., Kitayama J. (2008). Epigallocatechin gallate affects human dendritic cell differentiation and maturation. J. Allergy Clin. Immunol..

[131] Balez R., Steiner N., Engel M., Munoz S.S., Lum J.S., Wu Y., Wang D., Vallotton P., Sachdev P., O’Connor M. (2016). Neuroprotective effects of apigenin against inflammation, neuronal excitability and apoptosis in an induced pluripotent stem cell model of Alzheimer’s disease. Sci. Rep..

[132] Ginwala R., McTish E., Raman C., Singh N., Nagarkatti M., Nagarkatti P., Sagar D., Jain P., Khan Z.K. (2016). Apigenin, a Natural Flavonoid, Attenuates EAE Severity Through the Modulation of Dendritic Cell and Other Immune Cell Functions. J. Neuroimmune Pharmacol..

[133] Sadraei H., Asghari G., Khanabadi M., Minaiyan M. (2017). Anti-inflammatory effect of apigenin and hydroalcoholic extract of Dracocephalum kotschyi on acetic acid-induced colitis in rats. Res. Pharm. Sci..

[134] Manach C., Scalbert A., Morand C., Remesy C., Jimenez L. (2004). Polyphenols: Food sources and bioavailability. Am. J. Clin. Nutr..

[135] van Meeteren M.E., Teunissen C.E., Dijkstra C.D., van Tol E.A. (2005). Antioxidants and polyunsaturated fatty acids in multiple sclerosis. Eur. J. Clin. Nutr..

[136] Shukla S., Gupta S. (2010). Apigenin: A promising molecule for cancer prevention. Pharm. Res..

[137] Venigalla M., Gyengesi E., Munch G. (2015). Curcumin and Apigenin—Novel and promising therapeutics against chronic neuroinflammation in Alzheimer’s disease. Neural Regen. Res..

[138] Patel D., Shukla S., Gupta S. (2007). Apigenin and cancer chemoprevention: Progress, potential and promise (review). Int. J. Oncol..

[139] Bruno A., Siena L., Gerbino S., Ferraro M., Chanez P., Giammanco M., Gjomarkaj M., Pace E. (2011). Apigenin affects leptin/leptin receptor pathway and induces cell apoptosis in lung adenocarcinoma cell line. Eur. J. Cancer.

[140] Zhang J., Zhao L., Cheng Q., Ji B., Yang M., Sanidad K.Z., Wang C., Zhou F. (2018). Structurally Different Flavonoid Subclasses Attenuate High-Fat and High-Fructose Diet Induced Metabolic Syndrome in Rats. J. Agric. Food Chem..

[141] Feng X., Weng D., Zhou F., Owen Y.D., Qin H., Zhao J., Wen Y., Huang Y., Chen J., Fu H. (2016). Activation of PPARgamma by a Natural Flavonoid Modulator, Apigenin Ameliorates Obesity-Related Inflammation Via Regulation of Macrophage Polarization. EBioMedicine.

[142] Kalivarathan J., Chandrasekaran S.P., Kalaivanan K., Ramachandran V., Carani Venkatraman A. (2017). Apigenin attenuates hippocampal oxidative events, inflammation and pathological alterations in rats fed high fat, fructose diet. Biomed. Pharmacother..

[143] Malik S., Suchal K., Khan S.I., Bhatia J., Kishore K., Dinda A.K., Arya D.S. (2017). Apigenin ameliorates streptozotocin-induced diabetic nephropathy in rats via MAPK-NF-kappaB-TNF-alpha and TGF-beta1-MAPK-fibronectin pathways. Am. J. Physiol. Renal Physiol..

[144] Ai X.Y., Qin Y., Liu H.J., Cui Z.H., Li M., Yang J.H., Zhong W.L., Liu Y.R., Chen S., Sun T. (2017). Apigenin inhibits colonic inflammation and tumorigenesis by suppressing STAT3-NF-kappaB signaling. Oncotarget.

[145] Marquez-Flores Y.K., Villegas I., Cardeno A., Rosillo M.A., Alarcon-de-la-Lastra C. (2016). Apigenin supplementation protects the development of dextran sulfate sodium-induced murine experimental colitis by inhibiting canonical and non-canonical inflammasome signaling pathways. J. Nutr. Biochem..

[146] Mascaraque C., Gonzalez R., Suarez M.D., Zarzuelo A., Sanchez de Medina F., Martinez-Augustin O. (2015). Intestinal anti-inflammatory activity of apigenin K in two rat colitis models induced by trinitrobenzenesulfonic acid and dextran sulphate sodium. Br. J. Nutr..

[147] Jung U.J., Cho Y.Y., Choi M.S. (2016). Apigenin Ameliorates Dyslipidemia, Hepatic Steatosis and Insulin Resistance by Modulating Metabolic and Transcriptional Profiles in the Liver of High-Fat Diet-Induced Obese Mice. Nutrients.

[148] Li F., Lang F., Zhang H., Xu L., Wang Y., Zhai C., Hao E. (2017). Apigenin Alleviates Endotoxin-Induced Myocardial Toxicity by Modulating Inflammation, Oxidative Stress, and Autophagy. Oxid. Med. Cell. Longev..

[149] Zeng P., Liu B., Wang Q., Fan Q., Diao J.X., Tang J., Fu X.Q., Sun X.G. (2015). Apigenin Attenuates Atherogenesis through Inducing Macrophage Apoptosis via Inhibition of AKT Ser473 Phosphorylation and Downregulation of Plasminogen Activator Inhibitor-2. Oxid. Med. Cell. Longev..

[150] Wang Q., Zeng P., Liu Y., Wen G., Fu X., Sun X. (2015). Inhibition of autophagy ameliorates atherogenic inflammation by augmenting apigenin-induced macrophage apoptosis. Int. Immunopharmacol..

[151] Ren K., Jiang T., Zhou H.F., Liang Y., Zhao G.J. (2018). Apigenin Retards Atherogenesis by Promoting ABCA1-Mediated Cholesterol Efflux and Suppressing Inflammation. Cell. Physiol. Biochem..

[152] Yan X., Qi M., Li P., Zhan Y., Shao H. (2017). Apigenin in cancer therapy: Anti-cancer effects and mechanisms of action. Cell Biosci..

[153] Huang C., Wei Y.X., Shen M.C., Tu Y.H., Wang C.C., Huang H.C. (2016). Chrysin, Abundant in Morinda citrifolia Fruit Water-EtOAc Extracts, Combined with Apigenin Synergistically Induced Apoptosis and Inhibited Migration in Human Breast and Liver Cancer Cells. J. Agric. Food Chem..

[154] Nabavi S.M., Habtemariam S., Daglia M., Nabavi S.F. (2015). Apigenin and Breast Cancers: From Chemistry to Medicine. Anticancer Agents Med. Chem..

[155] Kashyapa D., Sharma A., Tulic H.S., Sakd K., Garge V.K., Buttarf H.S., Setzerg W.N., Sethih G. (2018). Apigenin: A natural bioactive flavone-type molecule with promising therapeutic function. J. Funct. Foods.

[156] Shukla S., Shankar E., Fu P., MacLennan G.T., Gupta S. (2015). Suppression of NF-kappaB and NF-kappaB-Regulated Gene Expression by Apigenin through IkappaBalpha and IKK Pathway in TRAMP Mice. PLoS ONE.

[157] Masuelli L., Benvenuto M., Mattera R., Di Stefano E., Zago E., Taffera G., Tresoldi I., Giganti M.G., Frajese G.V., Berardi G. (2017). In Vitro and In Vivo Anti-tumoral Effects of the Flavonoid Apigenin in Malignant Mesothelioma. Front. Pharmacol..

[158] Qian B.Z., Pollard J.W. (2010). Macrophage diversity enhances tumor progression and metastasis. Cell.

[159] Liao Y., Shen W., Kong G., Lv H., Tao W., Bo P. (2014). Apigenin induces the apoptosis and regulates MAPK signaling pathways in mouse macrophage ANA-1 cells. PLoS ONE.

[160] Mirzoeva S., Tong X., Bridgeman B.B., Plebanek M.P., Volpert O.V. (2018). Apigenin Inhibits UVB-Induced Skin Carcinogenesis: The Role of Thrombospondin-1 as an Anti-Inflammatory Factor. Neoplasia.

[161] Bauer D., Redmon N., Mazzio E., Soliman K.F. (2017). Apigenin inhibits TNFalpha/IL-1alpha-induced CCL2 release through IKBK-epsilon signaling in MDA-MB-231 human breast cancer cells. PLoS ONE.

[162] Lefort E.C., Blay J. (2013). Apigenin and its impact on gastrointestinal cancers. Mol. Nutr. Food Res..

[163] Kang H.K., Ecklund D., Liu M., Datta S.K. (2009). Apigenin, a non-mutagenic dietary flavonoid, suppresses lupus by inhibiting autoantigen presentation for expansion of autoreactive Th1 and Th17 cells. Arthritis Res. Ther..

[164] Lee J.H., Zhou H.Y., Cho S.Y., Kim Y.S., Lee Y.S., Jeong C.S. (2007). Anti-inflammatory mechanisms of apigenin: Inhibition of cyclooxygenase-2 expression, adhesion of monocytes to human umbilical vein endothelial cells, and expression of cellular adhesion molecules. Arch. Pharm. Res..

[165] Bailey S.L., Schreiner B., McMahon E.J., Miller S.D. (2007). CNS myeloid DCs presenting endogenous myelin peptides ’preferentially’ polarize CD4+ T(H)-17 cells in relapsing EAE. Nat. Immunol..

[166] Isaksson M., Ardesjo B., Ronnblom L., Kampe O., Lassmann H., Eloranta M.L., Lobell A. (2009). Plasmacytoid DC promote priming of autoimmune Th17 cells and EAE. Eur. J. Immunol..

[167] Ali R., Nicholas R.S., Muraro P.A. (2013). Drugs in development for relapsing multiple sclerosis. Drugs.

[168] Merad M., Sathe P., Helft J., Miller J., Mortha A. (2013). The dendritic cell lineage: Ontogeny and function of dendritic cells and their subsets in the steady state and the inflamed setting. Annu. Rev. Immunol..

[169] Mildner A., Jung S. (2014). Development and function of dendritic cell subsets. Immunity.

[170] Yoon M.S., Lee J.S., Choi B.M., Jeong Y.I., Lee C.M., Park J.H., Moon Y., Sung S.C., Lee S.K., Chang Y.H. (2006). Apigenin inhibits immunostimulatory function of dendritic cells: Implication of immunotherapeutic adjuvant. Mol. Pharmacol..

[171] Li X., Han Y., Zhou Q., Jie H., He Y., Han J., He J., Jiang Y., Sun E. (2016). Apigenin, a potent suppressor of dendritic cell maturation and migration, protects against collagen-induced arthritis. J. Cell. Mol. Med..

[172] Liu Y.F., Xue X.X., Li Z.Y., Wang J.P., Zhang Y.J. (2017). Effect of apigenin on dendritic cells maturation and function in murine splenocytes. Yao Xue Xue Bao.

[173] Li M., Zhang X., Zheng X., Lian D., Zhang Z.X., Ge W., Yang J., Vladau C., Suzuki M., Chen D. (2007). Immune modulation and tolerance induction by RelB-silenced dendritic cells through RNA interference. J. Immunol..

[174] Wu L., D’Amico A., Winkel K.D., Suter M., Lo D., Shortman K. (1998). RelB is essential for the development of myeloid-related CD8alpha- dendritic cells but not of lymphoid-related CD8alpha+ dendritic cells. Immunity.

[175] Platzer B., Jorgl A., Taschner S., Hocher B., Strobl H. (2004). RelB regulates human dendritic cell subset development by promoting monocyte intermediates. Blood.

[176] Jager A.K., Saaby L. (2011). Flavonoids and the CNS. Molecules.

[177] Ha S.K., Lee P., Park J.A., Oh H.R., Lee S.Y., Park J.H., Lee E.H., Ryu J.H., Lee K.R., Kim S.Y. (2011). Apigenin inhibits the production of NO and PGE2 in microglia and inhibits neuronal cell death in a middle cerebral artery occlusion-induced focal ischemia mice model. Neurochem. Int..

[178] Ren W., Qiao Z., Wang H., Zhu L., Zhang L. (2003). Flavonoids: Promising anticancer agents. Med. Res. Rev..

[179] Chun O.K., Chung S.J., Song W.O. (2007). Estimated dietary flavonoid intake and major food sources of U.S. adults. J. Nutr..

[180] Gradolatto A., Basly J.P., Berges R., Teyssier C., Chagnon M.C., Siess M.H., Canivenc-Lavier M.C. (2005). Pharmacokinetics and metabolism of apigenin in female and male rats after a single oral administration. Drug Metab. Dispos..

[181] Liu R., Zhang H., Yuan M., Zhou J., Tu Q., Liu J.J., Wang J. (2013). Synthesis and biological evaluation of apigenin derivatives as antibacterial and antiproliferative agents. Molecules.

[182] Ninomiya M., Tanaka K., Tsuchida Y., Muto Y., Koketsu M., Watanabe K. (2011). Increased bioavailability of tricin-amino acid derivatives via a prodrug approach. J. Med. Chem..

[183] Karim R., Palazzo C., Laloy J., Delvigne A.S., Vanslambrouck S., Jerome C., Lepeltier E., Orange F., Dogne J.M., Evrard B. (2017). Development and evaluation of injectable nanosized drug delivery systems for apigenin. Int. J. Pharm..

[184] Papay Z.E., Kallai-Szabo N., Balogh E., Ludanyi K., Klebovich I., Antal I. (2017). Controlled Release Oral Delivery of Apigenin Containing Pellets with Antioxidant Activity. Curr. Drug Deliv..

[185] Miyake M.M., Bleier B.S. (2015). The blood-brain barrier and nasal drug delivery to the central nervous system. Am. J. Rhinol. Allergy.

[186] Papay Z.E., Kosa A., Boddi B., Merchant Z., Saleem I.Y., Zariwala M.G., Klebovich I., Somavarapu S., Antal I. (2017). Study on the Pulmonary Delivery System of Apigenin-Loaded Albumin Nanocarriers with Antioxidant Activity. J. Aerosol. Med. Pulm. Drug Deliv..

